# Evolutionary analyses and expression patterns of TCP genes in Ranunculales

**DOI:** 10.3389/fpls.2022.1055196

**Published:** 2022-12-01

**Authors:** Catherine Damerval, Carmine Claudot, Martine Le Guilloux, Natalia Conde e Silva, Véronique Brunaud, Ludivine Soubigou-Taconnat, José Caius, Etienne Delannoy, Sophie Nadot, Florian Jabbour, Yves Deveaux

**Affiliations:** ^1^ Université Paris-Saclay, INRAE, CNRS, AgroParisTech, Génétique Quantitative et Evolution-Le Moulon, IDEEV, Gif-sur-Yvette, France; ^2^ Université Paris-Saclay, CNRS, INRAE, Univ Evry, Institute of Plant Sciences Paris-Saclay (IPS2), Orsay, France; ^3^ Université Paris-Saclay, CNRS, AgroParisTech, Ecologie Systématique Evolution, Orsay, France; ^4^ Institut de Systématique, Evolution, Biodiversité (ISYEB), Muséum National d’Histoire Naturelle, CNRS, Sorbonne Université, EPHE, Université des Antilles, Paris, France

**Keywords:** Ranunculales, *Nigella damascena*, TCP gene family, gene phylogeny, amino acid motifs, gene duplication

## Abstract

TCP transcription factors play a role in a large number of developmental processes and are at the crossroads of numerous hormonal biosynthetic and signaling pathways. The complete repertoire of TCP genes has already been characterized in several plant species, but not in any species of early diverging eudicots. We focused on the order Ranunculales because of its phylogenetic position as sister group to all other eudicots and its important morphological diversity. Results show that all the TCP genes expressed in the floral transcriptome of *Nigella damascena* (Ranunculaceae) are the orthologs of the TCP genes previously identified from the fully sequenced genome of *Aquilegia coerulea*. Phylogenetic analyses combined with the identification of conserved amino acid motifs suggest that six paralogous genes of class I TCP transcription factors were present in the common ancestor of angiosperms. We highlight independent duplications in core eudicots and Ranunculales within the class I and class II subfamilies, resulting in different numbers of paralogs within the main subclasses of TCP genes. This has most probably major consequences on the functional diversification of these genes in different plant clades. The expression patterns of TCP genes in *Nigella damascena* were consistent with the general suggestion that CIN and class I TCP genes may have redundant roles or take part in same pathways, while CYC/TB1 genes have more specific actions. Our findings open the way for future studies at the tissue level, and for investigating redundancy and subfunctionalisation in TCP genes and their role in the evolution of morphological novelties.

## Introduction

The TCP family of transcription factors is specific to Viridiplantae. Whether this gene family originated in the most recent common ancestor of Embryophyta (land plants) or in aquatic Streptophyta prior to the divergence of Zygnematophyta remains controversial (Navaud et al., 2007; Liu et al., 2019; Wang et al., 2022). Over the course of evolution, the number of TCP genes has increased markedly in seed plants and especially in angiosperms compared with other embryophytes (Navaud et al., 2007; Li, 2015; Liu et al., 2019; Wang et al., 2022). The TCP gene family was initially defined from structural homologies in the three founding gene members, *TEOSINTE-BRANCHED1* (*TB1*) in maize, *CYCLOIDEA* (*CYC*) in snapdragon and *PROLIFERATING CELL NUCLEAR ANTIGEN FACTOR1* (*PCF1*) and *PCF2* in rice (Luo et al., 1996; Doebley et al., 1997; Kosugi & Ohashi, 1997; Cubas et al., 1999). Their characteristic domain (the TCP domain) is a non-canonical basic helix-loop-helix (bHLH) domain (Cubas et al., 1999). Two classes of TCP transcription factors have been defined based on structural characteristics in the basic and second helix regions of the TCP domain. Class I proteins, first described in PCFs, are characterized by a 55-62 amino acid long TCP domain, with a four amino acid shorter basic region and a longer second helix than class II proteins. Class II proteins, such as CYC and TB1, are characterized by a 58-59 amino acid long TCP domain (Cubas et al., 1999; Aggarwal et al., 2010). While phylogenetic reconstructions are presently unable to resolve which of the two classes evolved first, they reveal that class I genes are less divergent from each other than class II genes. Indeed, two subfamilies of class II genes have been defined. The CIN subfamily, named after the snapdragon TCP gene *CINCINNATA* (*CIN*) (Nath et al., 2003), is present in all land plants. By contrast, the CYC/TB1 subfamily is found only in angiosperms, suggesting that this subfamily may have evolved from the CIN subfamily within class II (González-Grandío and Cubas, 2016; Liu et al., 2019).

It appears that TCP transcription factors bind to DNA as homo or heterodimers, mainly with members of the same class (Kosugi and Ohashi, 2002; Costa et al., 2005; Aggarwal et al., 2010; Danisman et al., 2013). TCP proteins recognize 6-10 bp motifs containing a GGNCC or GGNNCC core sequence (reviewed in González-Grandío and Cubas, 2016). More precisely, class I proteins bind to the consensus sequence GTGGGNCC and class II proteins to GTGGNCCC (Kosugi and Ohashi, 2002; Uberti-Manassero et al., 2013). Binding specificity appears to be determined mainly by specific amino acids in the basic region of the TCP domain, i.e. Gly at position 11 in class I and Asp at position 15 in class II (Kosugi and Ohashi, 1997; Aggarwal et al., 2010; Viola et al., 2012; González-Grandío and Cubas, 2016). A leucine-rich motif present in the second helix of the TCP domain could promote protein-protein interactions (Li, 2015). The arginine-rich R domain found in several class II proteins is predicted to form a coiled coil that could also promote protein-protein interactions (Cubas et al., 1999).

TCP transcription factors have been found to play a role in a large spectrum of developmental processes, such as floral symmetry, flowering time, leaf development and senescence, shoot branching, circadian clock and plant defense responses (Luo et al., 1996; Nath et al., 2003; Palatnik et al., 2003; Takeda et al., 2003; Crawford et al., 2004; Ori et al., 2007; Kim et al., 2008; Yuan et al., 2009; Giraud et al., 2010; Koyama et al., 2011; Hao et al., 2012; Aguilar-Martínez and Sinha, 2013; Juntheikki-Palovaara et al., 2014; Huang and Irish, 2015; Lucero et al., 2015; Koyama et al., 2017; Challa et al., 2018; Vadde et al., 2018; Challa et al., 2019; Li et al., 2019; Camoirano et al., 2020; Gastaldi et al., 2020; Zhang et al., 2020). In addition to their role in the transcriptional control of cell cycle genes (Kosugi and Ohashi, 1997; Gaudin et al., 2000; Nath et al., 2003), TCP proteins act in several regulatory networks including other transcription factors, miRNA-controlled pathways, and hormone biosynthesis and signaling pathways (Danisman et al., 2012; Das Gupta et al., 2014; Schommer et al., 2014; Nicolas and Cubas, 2016a; Nicolas and Cubas, 2016b; van Es et al., 2018; Zhou et al., 2018; Challa et al., 2019; Gastaldi et al., 2020). An increasing number of studies have revealed that the initial dichotomy of growth activator *vs* repressor role of class I *vs* class II genes is in fact much more complex, with both classes displaying antagonistic as well as synergistic actions in various growth processes (Nicolas and Cubas, 2016b; van Es et al., 2019).

Functional studies require knowledge of the complete repertoire of proteins in a multigene family such as the TCP family to track possible redundancy and investigate the specificity of action of its members. It is also important to understand the evolutionary relationship of gene family members and the large scale history of the gene family in question. Several complete genomes have been screened for the full repertoire of TCP genes, especially in core eudicots and monocots (e.g. Parapunova et al., 2014; Francis et al., 2016; Zheng et al., 2018; Huo et al., 2019; Jiu et al., 2019; Leng et al., 2019; Li et al., 2021; Zhao et al., 2021), but data is scarce in early diverging eudicots (Citerne et al., 2013; Liu et al., 2019). In this paper, we set out to characterize the gene repertoire and evolutionary history of the TCP family in the order Ranunculales, the sister group to all other eudicots. We took advantage of the available floral transcriptome of the Ranunculaceae species *Nigella damascena* L. to characterize expressed TCP genes, and we combined phylogenetic reconstruction with the presence and arrangement of conserved amino acid motifs as additional phylogenetic markers to (i) assess the orthology relationship of *N. damascena* genes with genes from the fully sequenced genome of another Ranunculaceae species *Aquilegia coerulea*, and (ii) reconstruct the evolutionary history of both class I and class II transcription factor genes in selected angiosperm species with fully sequenced genomes, as well as specifically within Ranunculales. Our results suggest that the fourteen transcripts characterized in *N. damascena* are the orthologs of the fourteen TCP genes found in the fully sequenced genome of *A. coerulea*, and that the evolutionary history of TCP genes differs between Ranunculales and core eudicots, with evidence of independent large scale (i.e. concerning high rank taxa) gene duplications. Finally, we characterized TCP gene expression profiles in aerial tissues of *N. damascena* and discuss the results in the light of repertoire complexity and developmental processes where TCP genes are known to play a role in model eudicot species.

## Material and methods

### TCP homologs in *Nigella damascena*

We mined the annotated floral reference transcriptome of *N. damascena* for TCP proteins, identified from homology with the TCP proteins in the inferred proteomes of *Arabidopsis thaliana* and/or *Aquilegia coerulea* as described in Deveaux et al. (2021). Seventeen contigs encoding peptides with homology with 12 *Arabidopsis* TCP proteins and/or 14 *Aquilegia* TCP proteins were found.

Primers were designed to specifically amplify these sequences from genomic DNA in order to validate the transcriptome assembly and detect the presence of introns (**Supplementary Table 1**). When necessary, semi-nested PCRs were done to amplify the 3’ end of the gene using an 18-base oligodT anchored with an A, G or C and two nested primers in the known 5’ part of the candidate gene (**Supplementary Table 1**). Amplification products were sequenced (Eurofin Genomics). The initial contig sequence was aligned with the amplified sequence and corrected if necessary. The comparison of the two sequences allowed us to detect the presence of introns (**Table 1**).

**Table 1:** Characteristics of TCP sequences in *Nigella damascena*, and their homologs in *Aquilegia coerulea*.

Contig name	*Nigella* gene name	Contig length (bp)	cds length (bp)	5’/3’ UTR minimum length (bp)	Intron (bp)	*Aquilegia* homolog	*Aquilegia* gene name
**class I**							
RN005256	*NdTCP7*	1607	777	95/735	–	Aqcoe3G008000	*AqTCP7*
RN006358	*NdTCP9-1*	1291	1098	157/17	–	Aqcoe7G051800	*AqTCP9-1*
RN065983	*NdTCP9-2*	1316	948	154/214	–	Aqcoe5G422600	*AqTCP9-2*
RN071097	NdTCP11	922	660	174/69	–	Aqcoe1G137200	*AqTCP11*
RN048267	*NdTCP15-1*	1665	1131	331/203	–	Aqcoe3G335300	*AqTCP15-1*
RN067927	*NdTCP15-2*	1447	1149	116/182	–	Aqcoe3G081500	*AqTCP15-2*
RN010851/2	*NdTCP20-1*	1497/1411	888	74/535	86 (3’ UTR)	Aqcoe5G347900	*AqTCP20-1*
RN015628/29	*NdTCP20-2*	1644/1748	921	239/484	104 (3’ UTR)	Aqcoe3G164300	*AqTCP20-2*
RN054028	*NdTCP22*	1611	1533 ^a^	nd/276	96 (3’ UTR)	Aqcoe6G015800	*AqTCP22*
**class II**							
RN066863/ RN057437	*NdCYL1*	333 477	1122 (1050) ^a,b^	nd/110	–	Aqcoe3G048600	*AqCYL1*
RN004309	*NdCYL2*	1314	1077	160/77	–	Aqcoe3G395500	*AqCYL2*
RN009083	*NdTCP2*	2766	1386	902/478	? (3'UTR)	Aqcoe4g007100	*AqTCP2*
RN002982	*NdTCP4*	2713	1191	1082/440	87 (3’ UTR)	Aqcoe1g240000	*AqTCP4*
RN003972	*NdTCP5*	1819	1176	356/287	378 (5’UTR)	Aqcoe3g370100	*AqTCP5*

^a^ 5’ end sequence obtained by chromosome walking; ^b^ two possible ATG codons; nd, not determined.

A genome walking strategy was used to obtain the full ORFs of TCP genes known from contigs with incomplete 5’ end coding sequences (*NdTCP22* and *NdCYL1*) using a protocol adapted from Balzergue et al. (2001). To summarize, 300 ng of genomic DNA were simultaneously digested by the PvII, EcoRI or DraI restriction enzyme and ligated to the asymmetric adapters in the T4 DNA ligase buffer (New England Biolabs). Specific nested primers were designed in the known 5’ region of the gene fragments and combined with nested adapter-specific primers. Amplification products were sequenced (Eurofin Genomics) and aligned to produce the consensus sequence. Gene-specific primers are listed in **Supplementary Table 1**. Genomic sequences were deposited in Genbank (accession numbers OP493852-865).

### Full repertoire of TCP proteins

Full repertoires of TCP genes were obtained from the annotated complete genomes of *Arabidopsis thaliana*, *Vitis vinifera*, *Nelumbo nucifera*, *Aquilegia coerulea*, *Oryza sativa* and *Amborella trichopoda*. For *Vitis* and *Nelumbo*, we combined data retrieved from PlantTFDB (v5.0, http://planttfdb.gao-lab.org/) and BLASTp analyses using *Arabidopsis* proteins as queries in the non-redundant database of Genbank (https://www.ncbi.nlm.nih.gov/genbank/). For *Oryza sativa* TCP transcription factor sequences were obtained from PlantTFDB and confirmed by the PFAM 03634 identifier at http://rice.plantbiology.msu.edu. For *Aquilegia coerulea*, we compared data from Plant TFDB and annotations in Phytozome (https://phytozome.jgi.doe.gov/). For *Amborella trichopoda*, 15 sequences were retrieved from Plant TFDB and Phytozome (**Supplementary Table 2**).

### Floral transcriptomes from four species of Ranunculaceae

*Aconitum napellus* and *Clematis stans* were grown at the Botanical garden of the French National Museum of Natural History, Paris, France. *Ficaria verna* and *Helleborus orientalis* were grown at the Jardin botanique de Launay, Orsay, France. Floral buds of various sizes covering the entire sequence of floral development for each species were harvested and immediately frozen in liquid nitrogen. Total RNA was extracted using the RNeasy Plant Mini Kit (Qiagen) with the additional DNAse I step according to the manufacturer’s instructions. Total RNA from each sample (one per species) was checked for integrity on an RNA_Nano chip using an Agilent 2100 bioanalyzer (Agilent Technologies, Waldbroon, Germany). Libraries were constructed with the TruSeq stranded mRNA library Prep kit (Illumina^®^, California, U.S.A.). They were paired-end (PE) sequenced with a read length of 100 bp using an Illumina HiSeq2000 at the Genoscope Laboratory (Evry, France). Lane distribution and barcoding gave approximately 25 to 35 million PE reads per sample. For each sample, raw data (fastq) were trimmed with Trimmomatic (Bolger et al., 2014) with a Phred Quality Score (Qscore) >20 and read lengths >30 bases. Ribosome sequences were removed with the sortMeRNA tool (Kopylova et al., 2012).

For each species, transcriptome assembly was made using Trinity (version 2.8.4, Grabherr et al., 2011) with default parameters and a kmer size of 32. Contigs that were smaller than 200 bases were removed. iAssembler (version 1.3, Zheng et al., 2011) was then used for scaffolding contigs and reducing redundancy, with –c option for strand specific assembly and 97% identity for sequence clustering, generating 53,205 (*A. napellus*), 35,842 (*H. orientalis*), 36,900 (*F. verna*) and 36,536 (*C. stans*) contigs (N50 respectively 1,147 bp, 1,529 bp, 1,434 bp and 1,330 bp). Proteomes were then generated using Transdecoder (version 5.3, Haas et al., 2013) with best orfs parameter, generating 31,831 (*A. napellus*), 25,647 (*H. orientalis*), 23,056 (*F. verna*) and 22,382 (*C. stans*) proteins. Data are available at https://doi.org/10.57745/2G1VCP.

### TCP sequences in Ranunculales

TCP sequences from Ranunculales were obtained from three sources. First, the 24 *Arabidopsis* TCP proteins were used as query for mining Genbank by tBLASTn, restricting the search to the seven Ranunculales families. Second, the 14 *Aquilegia coerulea* sequences were used as query for mining the Onekp database (https://db.cngb.org/onekp/) using tBLASTn, and sequences from Ranunculales species were selected. Third, we mined the floral transcriptomes of *Helleborus orientalis*, *Ficaria verna*, *Aconitum napellus* and *Clematis stans* (see above). The proteomes inferred from each of these transcriptomes were analyzed alongside the proteomes of *A. coerulea* and *N. damascena* using Orthofinder (Emms and Kelly, 2015; Emms and Kelly, 2019) with default options. Orthogroups that included at least one TCP protein from *A. coerulea* and/or *N. damascena* were kept, and transcripts of the homologous TCP proteins in *H. orientalis*, *F. verna*, *A. napellus* and *C. stans* were included in subsequent analyses.

The TCP sequence datasets obtained using the different strategies were then pruned to keep only unique amino acid sequences. In Papaveraceae, although we found sequences from several *Papaver* species in the databases, we retained only those of *P. somniferum*. The final dataset comprised in total 178 class II sequences and 145 class I sequences (**Supplementary Table 3A, B**). It is worth underlining that in most cases, these are expressed sequences and that the origin of multiple transcripts in a species is unknown (allelism, paralogy or alternative splicing).

### Phylogenetic analyses

Protein sequences were aligned using the online version of MAFFT (version 7) using the L-INS-i method (https://mafft.cbrc.jp/alignment/server/). Alignments were inspected and manually adjusted using BioEdit v.7.0.5 (Hall, 1999). The phylogeny of the full repertoire of TCP genes from seven angiosperm species (listed above) was reconstructed using protein sequences because of codon bias in the *Oryza sativa* nucleotide sequences. Phylogenetic analyses of TCP genes in Ranunculales were conducted on codon-based nucleotide alignments obtained from protein alignments using TranslatorX (http://translatorx.co.uk/, Abascal et al., 2010). Regions where primary homology could not be confidently established were manually removed from the alignments. Alignments were deposited on dryad (doi:10.5061/dryad.zcrjdfngw).

Phylogenetic analyses were done using PhymL (http://www.atgc-montpellier.fr/phyml/) with the automatic model selection by SMS option (Lefort et al., 2017); branch support was determined by the aLRT SH-like method (Guindon et al., 2010).

### Search for protein motifs

MEME in the MEME suite v5.3.3 (https://meme-suite.org) was used to identify conserved amino acid motifs in TCP class I and class II sequences. Default options were used except for the motif maximum width, which was fixed at 60 and 55 for class II and class I, respectively, and the number of motifs, which was fixed at 10 for both classes. Motifs are named as follows: Mx=motif x; I/II (superscript)=TCP class; R/nothing (subscript)=Ranunculales/full repertoire analysis. The presence and arrangement of conserved amino acid motifs along the whole length of the proteins were used to add support to the different groups identified in the phylogeny.

### TCP expression patterns in *N. damascena*

Seeds derived from six generations of selfing of a heterozygous plant from a commercial seed lot (Royal Fleur) were sown in a growth chamber under controlled conditions (18h day at 25°C, 6h night at 16°C). Plants were arranged in three replicates of 5 individuals. When the terminal bud was 12-13.5 mm in diameter, plants were harvested as follows: bracts, sepals, petals, stamens and carpels were dissected from the terminal flower, and the second lower bud (7-8 mm diameter), stem internodes and mature cauline leaves were collected separately (**Supplementary Figure 5**). All tissues were frozen in liquid nitrogen and stored at -80°C until RNA extraction.

For each organ sample and replicate, total RNA was extracted using the RNeasy Plant Mini Kit (Qiagen) with the additional DNAse I step according to the manufacturer’s instructions. An additional DNAse step was done before single stranded cDNAs were produced using SuperScriptII reverse transcriptase (Invitrogen) and a polyT primer. DNA contamination was excluded by performing no-RT negative controls using *ACTIN* specific primers (**Supplementary Table 1**). Each gene was amplified with specific primers, a specific annealing temperature and a specific number of cycles (**Supplementary Table 4**). *ACTIN* was used as a loading reference for comparison among samples.

## Results

### Characterization of TCP transcription factors in the floral transcriptome of *Nigella damascena*

Sixteen contigs encoding peptides with homology with 12 *Arabidopsis thaliana* and 14 *Aquilegia coerulea* TCP proteins were found in the annotated *N. damascena* floral transcriptome (Deveaux et al., 2021). An additional contig encoding a peptide that was only homologous to an *A. coerulea* TCP protein was also identified.

Genomic sequences corresponding to the *N. damascena* contigs were amplified to check the transcriptome assembly and determine the presence of introns. *N. damascena* sequences were then aligned and compared with their homologs in the *A. coerulea* genome (**Table 1**). Seven validated contigs (renamed *NdTCP7, NdTCP9-1, NdTCP9-2, NdTCP11, NdTCP15-1, NdTCP15-2* and *NdCYL2*) had complete coding sequences, 5’ and 3’ UTR of variable lengths and an absence of introns, like in their homologs in *A. coerulea*. The *NdTCP22* sequence was interrupted by an intron in the 3’ UTR, like its closest homolog in *A. coerulea* (Aqcoe6G015800, renamed as *AqTCP22*). Contig pairs (*NdTCP20-1/NdTCP20-2*) were homologous to Aqcoe5G347900 and Aqcoe3G164300, respectively, both of which were annotated as TCP20-like in Phytozome (*AqTCP20-1/AqTCP20-2*). Gene annotation revealed the presence of an intron in the 3’UTR of these genes in *A. coerulea*. In *N. damascena*, the members of each pair differed by an indel in the 3’ UTR, corresponding to alternative splicing of the intron. *NdTCP2* and *NdTCP4* had long 5’ UTRs (902 bp and 1,082 bp, respectively) and complete coding sequences. However, the 3’ UTR of *NdTCP2* could not be completely verified, and this may be due to the presence of a large intron, which is found in its closest homolog in *A. coerulea* (Aqcoe4g007100, *AqTCP2*). *NdTCP5* had both 5’ and 3’UTRs, with an intron detected in the 5’UTR, like in *A. coerulea* Aqcoe3g370100 (*AqTCP5*). The sequence for *NdCYL1* was re-assembled from two contigs that aligned with the 5’ and 3’ parts of Aqcoe3g048600 (*AqCYL1*), corresponding to one Ranunculaceae CYC-like homolog (RanaCYL1, Jabbour et al., 2014). The sequence was extended upstream by genome walking to obtain the complete coding sequence. No intron was found, unlike *AqCYL1* that has three introns interrupting the coding sequence. In total, we validated and fully sequenced 14 transcribed genes encoding 14 TCP proteins in *N. damascena* (**Table 1**).

### Relationships of *N. damascena* TCP proteins in the context of the evolutionary history of TCP proteins in angiosperms

To assess the orthology of *N. damascena* sequences with TCP genes from other angiosperms, we reconstructed the phylogeny of TCP proteins of selected angiosperm species with full genomes. The full inferred proteome of *Arabidopsis* comprises 24 TCP transcription factors, of which 13 belong to class I and 11 to class II (Cubas et al., 1999; Martín-Trillo & Cubas, 2010). In rice (*Oryza sativa* ssp *japonica*), we found 23 transcription factors corresponding to 21 different proteins in PlantTFDB, of which 11 belong to class II and 10 to class I. We found 11 *Amborella*, 7 *Nelumbo*, 9 *Vitis*, and 5 *Aquilegia* TCP proteins belonging to class II, and 9 *Aquilegia*, 9 *Vitis*, 9 *Nelumbo* and 6 *Amborella* proteins belonging to class I.

**Figure 1:**
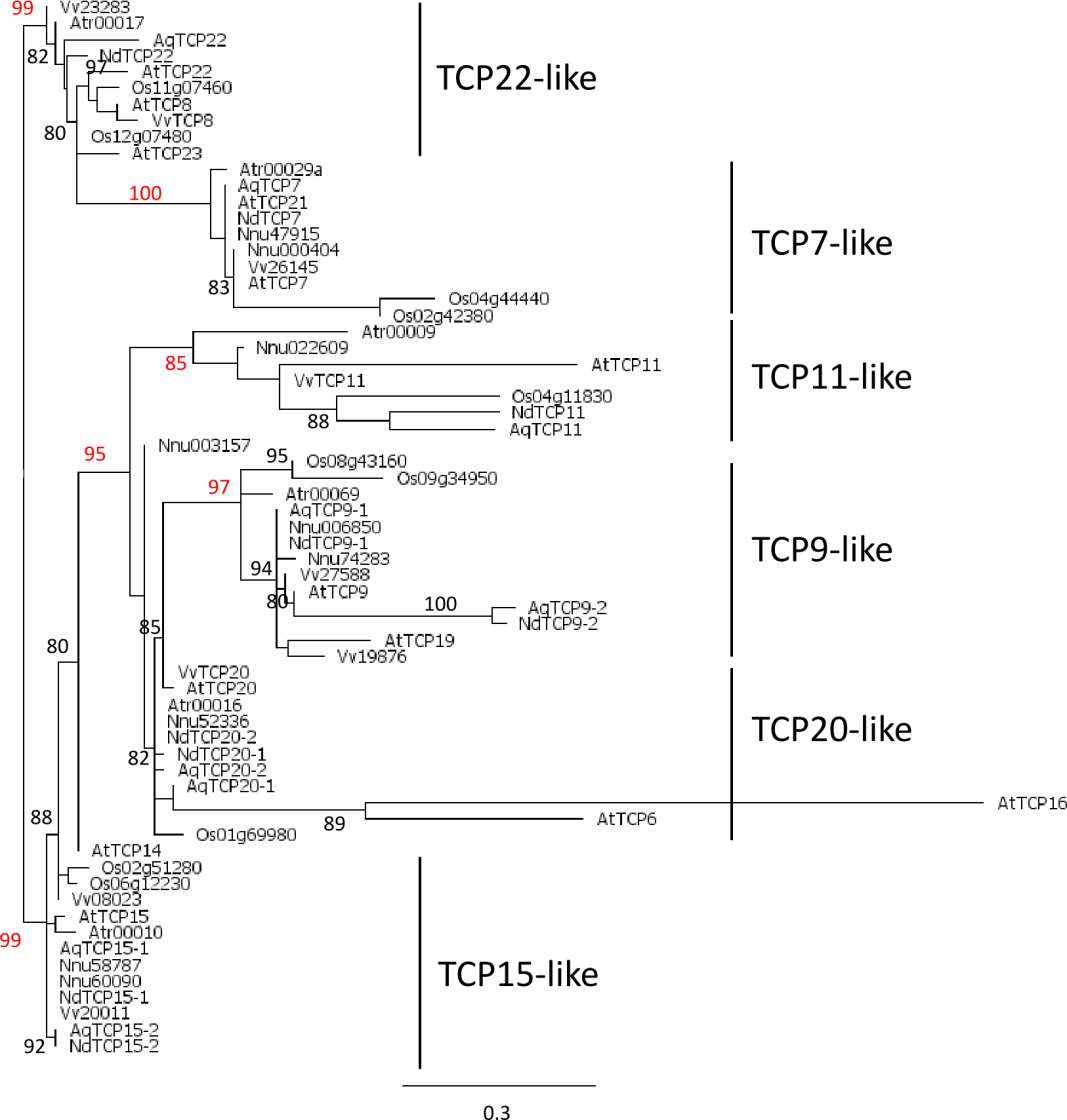
Phylogeny of class I TCP proteins in the complete inferred proteomes of *Amborella trichopoda* (Atr), *Arabidopsis thaliana* (At), *Vitis vinifera* (Vv), *Nelumbo nucifera* (Nnu), *Aquilegia coerulea* (Aq), *Oryza sativa* (Os), and the nine proteins deduced from the floral trancriptome of *Nigella damascena* (Nd). Maximum likelihood reconstruction was done with PhyML based on the alignment of 69 amino acid positions that included the TCP domain. Branch support values above 80% are indicated (a-LRT x100) with those discussed in the text in red.

Phylogenetic analysis of class I TCP proteins, based on 69 amino acid positions, revealed high support (a-LRT≥0.80) for several clades (**Figure 1**). One clade (a-LRT 0.99) comprised a paraphyletic group that we called TCP22-like, from the name of one of its members in *Arabidopsis*, and a monophyletic group (a-LRT 1.0), TCP7-like. Another clade (a-LRT 0.95) comprised two well supported sub-clades, TCP11-like (a-LRT 0.85) and TCP9-like (a-LRT 0.97) and a paraphyletic group (including Nnu003157), TCP20-like. Two *Arabidopsis* genes in this group, AtTCP6 and AtTCP16, were highly divergent from all other sequences and possibly grouped together because of long branch attraction. A last set of sequences, which share motif M^I^3 upstream of the TCP domain and for the most part motifs M^I^5 and M^I^6, was defined as TCP15-like (**Supplementary Table 5A**). While TCP11-like sequences were not characterized by any specific motif other than M^I^1 (the TCP domain) and M^I^2, the other clades had additional specific motifs (e.g. M^I^10 in TCP9-like, M^I^7 in TCP20-like) or specific combinations and order of motifs (**Supplementary Table 5A**). The M^I^2 motif, which has been identified as a characteristic motif of class I proteins (Aggarwal et al., 2010), flanks the TCP domain and is lacking only in the *Arabidopsis* proteins AtTCP6, AtTCP11 and AtTCP16 (**Supplementary Table 5A**). Each of the six groups we have defined comprised one protein sequence from *Amborella* and at least one protein sequence from each of the other species, with the exception of *Nelumbo* (which does not have a TCP22-like ortholog), supporting the hypothesis that the same number of class I paralogs existed in the common ancestor of angiosperms.

**Figure 2:**
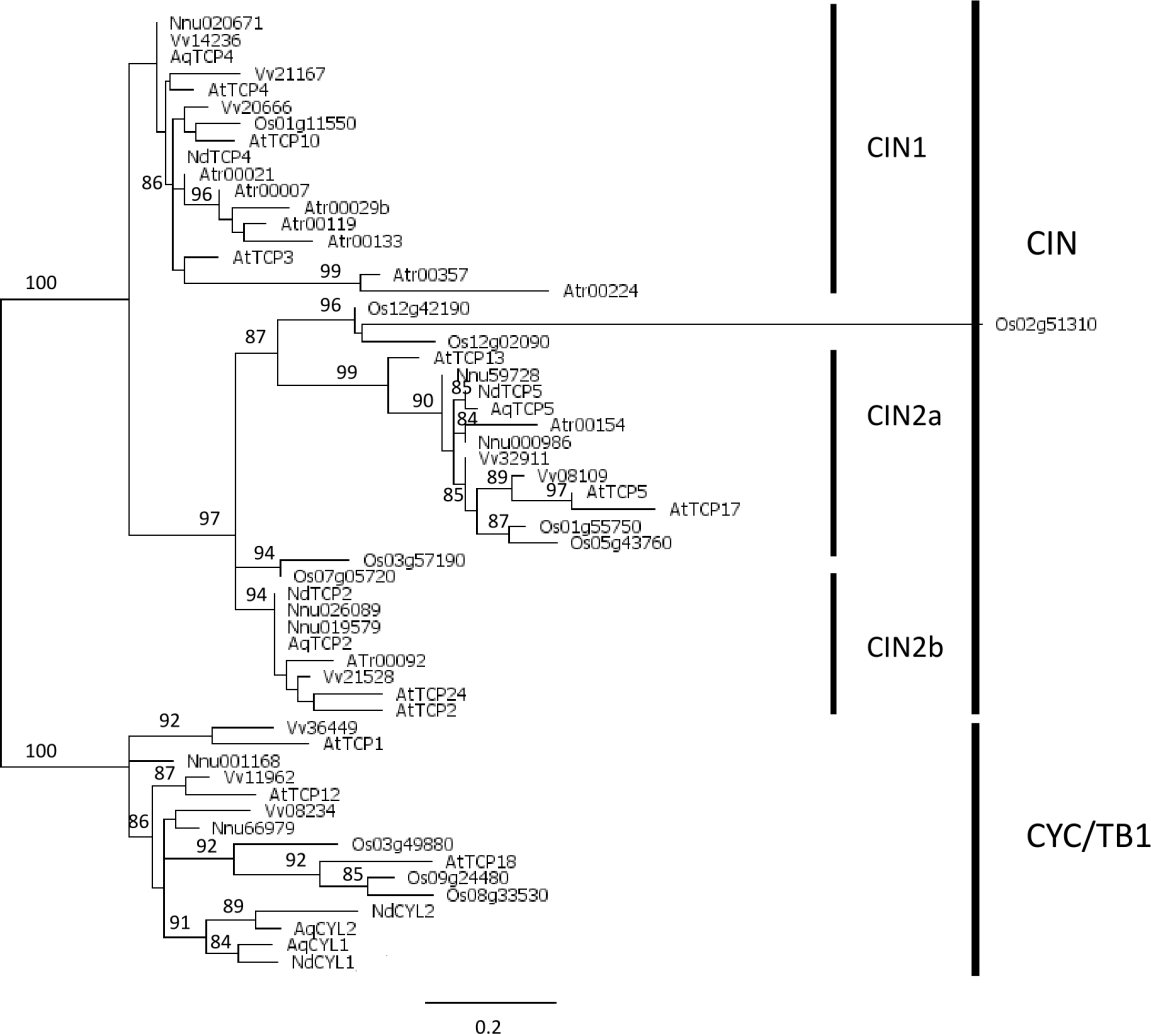
Phylogeny of class II TCP proteins in the complete inferred proteomes of *Amborella trichopoda* (Atr), *Arabidopsis thaliana* (At), *Vitis vinifera* (Vv), *Nelumbo nucifera* (Nnu), *Aquilegia coerulea* (Aq), *Oryza sativa* (Os), and the five proteins deduced from the floral trancriptome of *Nigella damascena* (Nd). Maximum likelihood reconstruction was done with PhyML and based on the alignment of 61 amino acid positions that included the TCP domain. Branch support values above 80% are indicated (a-LRT x100).

The phylogenic analysis of class II proteins (61 amino acid positions) recovered the two previously characterized CYC/TB1 and CIN sub-classes (**Figure 2**). At least one protein sequence from each species except *Amborella* was found in the CYC/TB1 sub-class. Within this clade, some branches were well-supported, for example the two paralogous lineages previously described in Ranunculaceae (AqCYL1/NdCYL1 and AqCYL2/NdCYL2, respectively, Citerne et al., 2013; Jabbour et al., 2014), two pairs of *Arabidopsis* and *Vitis* proteins belonging to the previously described CYC2 (AtTCP1 and Vv36449) and CYC3 (AtTCP12 and Vv08234) clades (Howarth and Donoghue, 2006), and a small clade formed by AtTCP18 and three rice proteins. All proteins in this clade except Os08g33530 and Os09g24480 had an R domain (M^II^2 motif, **Supplementary Table 5B**). Within the CIN sub-class, the CIN1 clade was weakly supported (a-LRT 0.74) while the CIN2 clade was well supported (a-LRT 0.97). The two sub-classes each have a specific motif upstream of the TCP domain (M^II^1), i.e. M^II^4 for CIN1 and M^II^6 for CIN2 (**Supplementary Table 5B**). The CIN1 clade was poorly resolved, and comprised one rice sequence, three *Arabidopsis* (AtTCP3, AtTCP4 and AtTCP10) and three *Vitis* sequences, one sequence from each early diverging eudicot species and seven *Amborella* sequences, which were more closely related to each other than to sequences of the other species. There are two well supported clades, which we called CIN2a (a-LRT 0.87) and CIN2b (a-LRT 0.94), within the CIN2 clade, each containing a single *Amborella* sequence. CIN2b sequences differed from CIN2a sequences by the presence of several amino acid motifs (e.g. M^II^2 (the R domain), M^II^9 and to a lesser extent M^II^7). The M^II^9 motif is found in the closely related *Oryza* Os03g57190 and Os07g05720 (M^II^2 is found in the latter), suggesting these sequences are closer to CIN2b; by contrast, the triplet including Os12g02090 has a low number of characteristic motifs so that its status remains uncertain (**Supplementary Table 5B**). As for early diverging eudicots, each CIN2 clade contained two paralogs from *Nelumbo* and one sequence from the two Ranunculaceae species.

In every clade described above, the phylogeny generally supported the orthology between one *N. damascena* sequence and one *A. coerulea* sequence, suggesting we obtained the full repertoire of TCP proteins from our *N. damascena* floral transcriptome.

### Evolutionary history of TCP transcription factors in Ranunculales

#### Phylogenetic analysis of class I TCP genes

**Figure 3:**
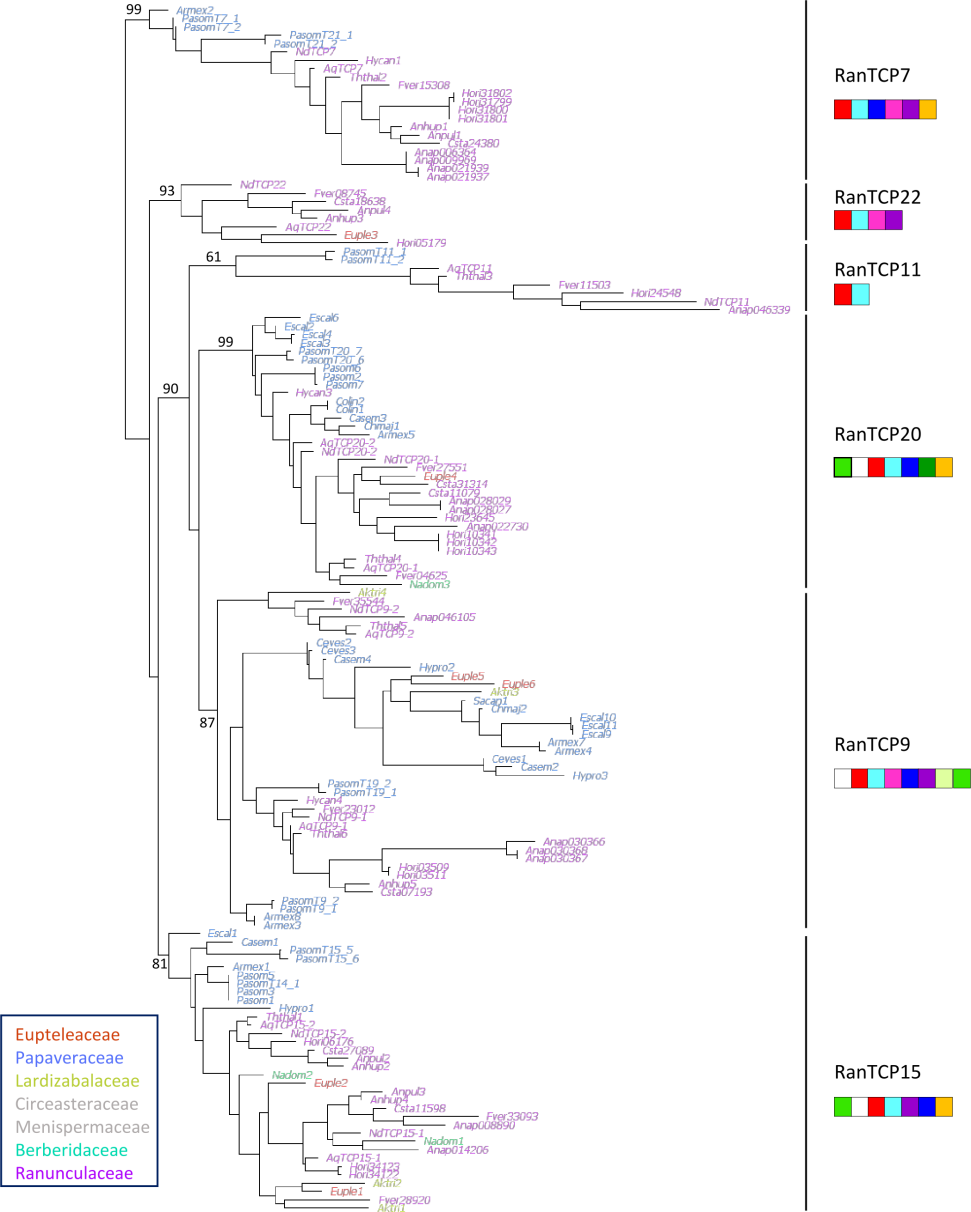
Phylogeny of 145 class I TCP coding nucleotide sequences from Ranunculales. Ranunculales families were color-coded as indicated (no sequences were available for Circeasteraceae and Menispermaceae, in grey). Support values (a-LRT x100) are given for the main branches, corresponding to the six groups identified in **Figure 1**. The specific combinations of amino acid motifs that characterize the six groups are drawn under each group name. A motif is represented if it is found in at least half of the sequences in that group. Red: ${\text{M}}_{\text{R}}^{\text{I}}1$ ; light blue: ${\text{M}}_{\text{R}}^{\text{I}}2$ ; mid green: ${\text{M}}_{\text{R}}^{\text{I}}3$ ; violet: ${\text{M}}_{\text{R}}^{\text{I}}4$ ; yellow: ${\text{M}}_{\text{R}}^{\text{I}}5$ ; dark green: ${\text{M}}_{\text{R}}^{\text{I}}6$ ; dark blue: ${\text{M}}_{\text{R}}^{\text{I}}7$ ; pink: ${\text{M}}_{\text{R}}^{\text{I}}8$ ; white: ${\text{M}}_{\text{R}}^{\text{I}}9$ ; yellow green: ${\text{M}}_{\text{R}}^{\text{I}}10$ (motifs defined in **Supplementary Figure 2**).

145 sequences belonging to 21 genera from five families out of the seven that are recognized in Ranunculales were retrieved from databases as well as from our own transcriptomic data. The phylogenetic analysis was based on an alignment of 207 nucleotide sites that included the TCP domain (**Figure 3**). The six groups of class I TCP proteins described above were recovered in the analysis of sequences from Ranunculales, each group containing sequences from species belonging to at least two different families. These groups were supported by specific combinations and order of protein motifs (**Figure 3**, **Supplementary Figure 2**). Most of these motifs were also detected in the complete repertoire analysis (see **Supplementary Table 5A** for correspondence of motifs). As expected, the ${\text{M}}_{\text{R}}^{\text{I}}1$ (TCP domain) and ${\text{M}}_{\text{R}}^{\text{I}}2$ (characteristic of class I TCP proteins) motifs were found in all sequences. The ${\text{M}}_{\text{R}}^{\text{I}}3$ motif was found upstream of the TCP domain in the RanTCP15 and RanTCP20 clades (a-LRT 0.99 and 0.81, respectively), whereas it was found downstream of the TCP domain in the RanTCP9 clade (a-LRT 0.89). The ${\text{M}}_{\text{R}}^{\text{I}}10$ motif was present in most RanTCP9 sequences, while the ${\text{M}}_{\text{R}}^{\text{I}}6$ motif was characteristic of the RanTCP20 clade. The RanTCP7 clade (a-LRT 0.99) was not characterized by any specific motif, but rather by the combination of five motifs in a specific order ( ${\text{M}}_{\text{R}}^{\text{I}}7$ , ${\text{M}}_{\text{R}}^{\text{I}}8$ , ${\text{M}}_{\text{R}}^{\text{I}}4$ , ${\text{M}}_{\text{R}}^{\text{I}}9$ and ${\text{M}}_{\text{R}}^{\text{I}}5$ ). A poorly supported group (RanTCP11, a-LRT 0.61) with long branches aggregated sequences without specific motifs except the characteristic ${\text{M}}_{\text{R}}^{\text{I}}1$ and ${\text{M}}_{\text{R}}^{\text{I}}2$ motifs. The sixth clade (RanTCP22) was well supported (a-LRT 0.93) and included sequences characterized by a low number of conserved amino acid motifs in addition to ${\text{M}}_{\text{R}}^{\text{I}}1$ and ${\text{M}}_{\text{R}}^{\text{I}}2$ . Additional phylogenetic analyses of each of the six groups rooted with the closest homologous sequences from *Nelumbo* (or *Vitis* for RanTCP22), showed that sequence relationships were generally congruent with species relationships (**Supplementary Figures 1A-F**). There was a duplication in the RanTCP20 clade in Ranunculaceae but the timing of this duplication in the evolutionary history of Ranunculales could not be ascertained due to insufficient sampling of the other families. A duplication was observed in the RanTCP15 clade, which could have taken place after the divergence of Papaveraceae. In the RanTCP9 clade, a duplication after the divergence of Eupteleaceae can be hypothesized (**Supplementary Figures 1A-F**).

#### Phylogenetic analysis of class II TCP genes

**Figure 4:**
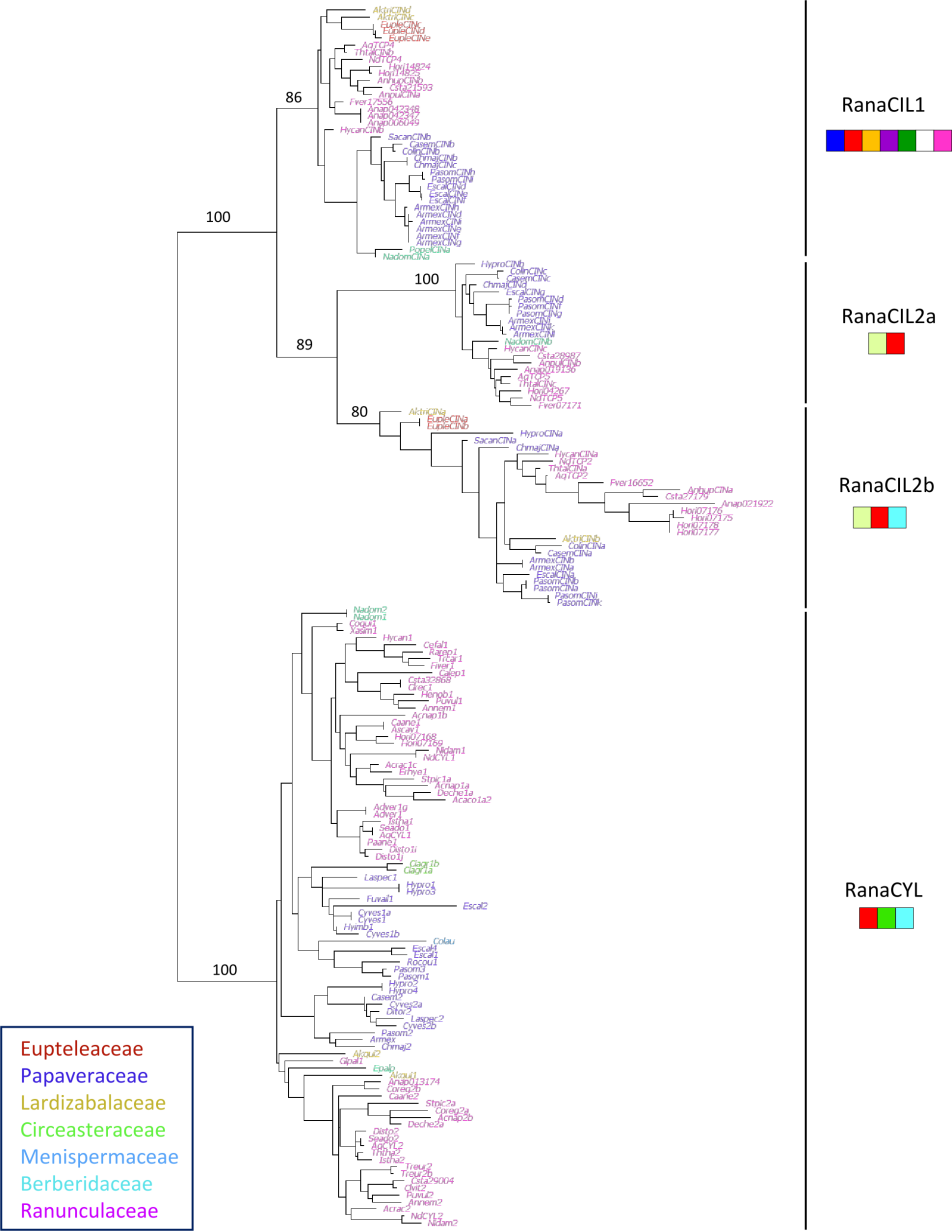
Phylogeny of 173 class II TCP coding sequences from Ranunculales. Ranunculales families were color-coded as indicated. Support values (a-LRT x100) are given for the main branches, corresponding to clades identified in **Figure 2**. The specific combinations of amino acid motifs that characterize the four clades are drawn under each clade name. A motif is represented if it is found in more than half of the sequences in that clade. Red: ${\text{M}}_{\text{R}}^{\text{II}}1$ ; light blue: ${\text{M}}_{\text{R}}^{\text{II}}2$ ; mid green: ${\text{M}}_{\text{R}}^{\text{II}}3$ ; violet: ${\text{M}}_{\text{R}}^{\text{II}}4$ ; yellow: ${\text{M}}_{\text{R}}^{\text{II}}5$ ; dark green: ${\text{M}}_{\text{R}}^{\text{II}}6$ ; dark blue: ${\text{M}}_{\text{R}}^{\text{II}}7$ ; pink: ${\text{M}}_{\text{R}}^{\text{II}}8$ ; white: ${\text{M}}_{\text{R}}^{\text{II}}9$ ; yellow green: ${\text{M}}_{\text{R}}^{\text{II}}10$ (motifs defined in **Supplementary Figure 4**).

We compiled 178 sequences of class II TCP genes from 52 genera representing all seven families within Ranunculales. Similarly to class I, sequences from Ranunculaceae and Papaveraceae were over-represented. Phylogenetic reconstruction was based on a 199 nucleotide site alignment that included the TCP domain and its flanking regions, available in 173 out of the 178 sequences (**Figure 4**). The two subfamilies CYC/TB1 and CIN were well supported. Within the CIN clade, the three subfamilies described above were recovered, called here RanaCIL1, RanaCIL2a and RanaCIL2b. A characteristic difference between RanaCIL1 and RanaCIL2 was the presence of an alternative motif immediately upstream of the TCP domain ( ${\text{M}}_{\text{R}}^{\text{II}}1$ ), i.e. ${\text{M}}_{\text{R}}^{\text{II}}7$ for RanaCIL1 and ${\text{M}}_{\text{R}}^{\text{II}}10$ for RanaCIL2 (**Figure 4**; **Supplementary Figure 4**, see also **Supplementary Table 5B** for correspondence of motifs with the full repertoire analysis). RanaCYL sequences were characterized by having an R domain ( ${\text{M}}_{\text{R}}^{\text{II}}2$ ) and an ECE motif ( ${\text{M}}_{\text{R}}^{\text{II}}3$ ). Two sequences in the RanaCIL2a clade had an R domain in addition to the TCP domain and the ${\text{M}}_{\text{R}}^{\text{II}}10$ motif. RanaCIL1 sequences had the largest number of conserved protein motifs (**Figure 4**). Detailed phylogenetic analyses of the RanaCIL clade showed that sequence relationships were congruent with species relationships, with some species specific gene duplications (**Supplementary Figures 3A-C**). Within the RanaCYL clade, a gene duplication may have taken place after the divergence of Eupteleaceae, which has two paralogs that are probably the result of a lineage specific duplication (**Supplementary Figures 3A-D**).

### Expression patterns of TCP genes in aerial tissues of *Nigella damascena*

Semi-quantitative RT-PCR was used to obtain an overview of the expression profile of the 14 *N. damascena* TCP genes in aerial tissues of plants close to blooming (**Supplementary Figure 5**).

**Figure 5:**
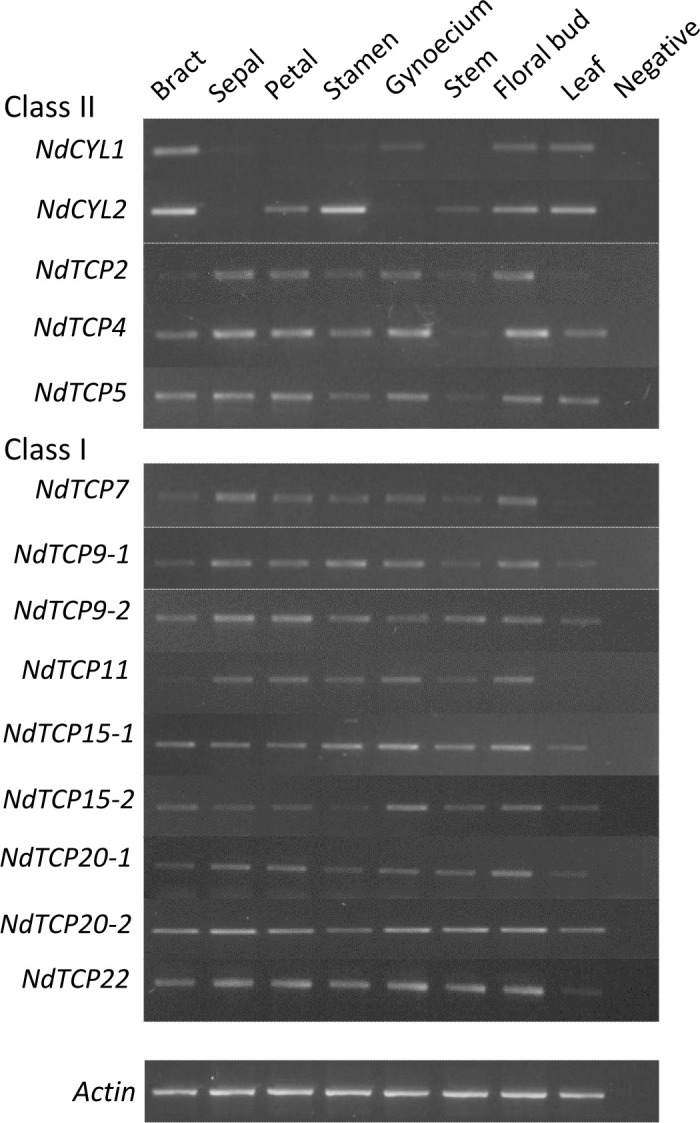
Expression patterns of all *Nigella damascena* TCP genes in eight aerial organs.

Results were generally consistent among the three biological replicates (**Supplementary Figure 6**). All TCP genes were expressed in most to all organs, with the exception of the two *NdCYLs* (**Figure 5**). Indeed, in the floral organs, *NdCYL2* was more strongly expressed in petals and stamens whereas *NdCYL1* was relatively more expressed in the gynoecium. The three *NdCIL* genes (*NdTCP2*, *NdTCP4* and *NdTCP5*) had a more even expression pattern among organs, with low expression in bracts and leaves for *NdTCP2*. *NdTCP2* and *NdTCP5* were comparatively less expressed in stamens than in other dissected floral organs. Similarly, the expression patterns of class I genes were generally similar across all organs. The expression of *NdTCP7* and *NdTCP11* in bracts and leaves was weak compared with the other organs. Among dissected floral organs, *NdTCP9-2* appeared less expressed in the reproductive organs than in the perianth organs, and expression of *NdTCP15-2* was highest in the gynoecium (**Figure 5**; **Supplementary Figure 6**).

## Discussion

The Ranunculales appears to be the first order to have diverged within the eudicots. It stands out by its remarkable morphological diversity in both vegetative and reproductive traits. Its phylogenetic position as sister to all other eudicots makes it an important group for investigating the origin of morphological diversity in a comparative framework, in particular compared with other major angiosperm groups such as the successful monocots and core eudicots (Becker, 2016; Damerval and Becker, 2017; Zhao et al., 2018; Pabón-Mora et al., 2020; Martínez-Gómez et al., 2021). TCP transcription factor genes have been shown to be major actors of plant development since the initial characterization of the family (Cubas et al., 1999). While this gene family has been characterized in many core eudicot and monocot species, no systematic investigation has been conducted in early diverging eudicots and Ranunculales in particular. A comprehensive survey of TCP genes in Ranunculales will help clarify the evolutionary history of this gene family and understand the functional evolution of TCP genes in angiosperms as a whole. The recent availability of genomic resources, i.e. the full genome of *Aquilegia coerulea* (Filiault et al., 2018) and our floral transcriptome of *Nigella damascena* (Deveaux et al., 2021), allowed us to characterize which could be the complete repertoire of both class I and class II TCP genes in *N. damascena*. Using phylogenetic reconstructions combined with analyses of conserved protein motifs, we propose a new evolutionary hypothesis for class I TCP transcription factors in angiosperms. Within the eudicot clade, we establish that the gene duplication history of the TCP family occurred independently in Ranunculales and core eudicots.

Accessing the full repertoire of TCP genes in a given species is an important step to assess the involvement of members of this family in developmental processes, as the analysis of individual genes may be blurred by redundancy (Danisman et al., 2013). Protein relatedness, as inferred from phylogenetic analyses, and similar spatio-temporal regulation are essential components of redundancy. We determine the expression patterns of all TCP genes in an early diverging eudicot species, *Nigella damascena*. We find that at the organ level, CIN and class I TCP genes have similar expression patterns, which could suggest redundant roles or involvement in the same pathways, as was found in some core eudicot species. By contrast, CYC/TB1 genes seem to have more differentiated expression domains.

### Evolutionary history of class I TCP genes and independent duplications in Ranunculales and core eudicots

The complete repertoire of class I proteins amounted to 6 in *Amborella*, 13 in *Arabidopsis*, 10 in *Oryza* and 9 in *Vitis*, *Nelumbo*, *Aquilegia* and *Nigella*. Phylogenetic analysis showed that class I proteins are more conserved than class II proteins, as previously observed (Martín-Trillo and Cubas, 2010; Mondragón-Palomino and Trontin, 2011; Liu et al., 2019). Six groups of class I genes were identified in angiosperms on the phylogenetic tree, each of which contained a single sequence from *A. trichopoda*. The circumscription of these groups was confirmed by the specific association of conserved protein motifs. These results suggest that there were six paralogous copies of class I TCP genes in the common ancestor of angiosperms. Only one copy of TCP11-like was found in all species sampled. In the TCP22-like and TCP7-like groups, gene duplications were identified in *Oryza*, *Vitis* and *Arabidopsis*, but only one gene was found in Ranunculaceae. Duplications were also observed in all species in the TCP9-like, TCP20-like and TCP15-like groups, which were difficult to place reliably in the evolutionary history of class I genes because of low resolution (**Figure 1**). These six TCP groups were confirmed in the analysis of Ranunculales class I sequences, and provided a framework for more comprehensive analyses. The phylogenetic analyses of Ranunculales sequences in these groups rooted with sequences of a non-Ranunculales species support the occurrence of Ranunculales specific duplications, prior to the divergence of Ranunculaceae for TCP20-like, and possibly after the divergence of Eupteleaceae for TCP15-like and perhaps also for TCP9-like (**Supplementary Figures 1D-F**). These analyses suggest that although early diverging eudicots and core eudicots have the same number of class I paralogs [with the exception of *Arabidopsis*, which, unlike *Vitis*, has undergone additional whole genome duplications (Vision et al., 2000)], their duplication history is different. Independent whole genome duplications in core eudicots and at some points during the evolutionary history of the Ranunculales could account for these results (Cui et al., 2006; Jiao et al., 2012; Shi and Chen, 2020). The duplicate evolutionary history and therefore their potential functional specialization, were mostly taxon-specific.

### Evolutionary history of class II TCP genes and low copy number in the CIN clade in Ranunculales

The classical subdivision of class II proteins into the CYC/TB1 and CIN clades was recovered (Martín-Trillo and Cubas, 2010; Mondragón-Palomino and Trontin, 2011; Liu et al., 2019). While CYC/TB1 sequences are specific to angiosperms (Navaud et al., 2007; Horn et al., 2014; Liu et al., 2019), closely related sequences have recently been found in Gymnosperms (Wang et al., 2022). We confirmed the absence of CYC/TB1 in *Amborella trichopoda* (Liu et al., 2019; Wang et al., 2022), but our analysis could not determine whether CYC/TB1 had been lost in this species or if the CYC/TB1 lineage originated after the divergence of *Amborella* from the rest of angiosperms. Two to three paralogs were found in the complete repertoire of the other species sampled. The close phylogenetic relationship between the rice sequences and AtTCP18 suggests that only one CYC/TB1 gene was present in the common ancestor of the monocots and eudicots, with further taxon-specific duplications. Several studies have revealed the complex history of this clade in monocots (Mondragón-Palomino and Trontin, 2011; Bartlett and Specht, 2011; Preston and Hileman, 2012). The core eudicot specific CYC1, CYC2 and CYC3 clades that have been described previously (Howarth and Donoghue, 2006) may have been generated by the genome triplication which occurred in the common ancestor of this large clade. Independent duplications were reported in Ranunculales and in *Nelumbo* (Citerne et al., 2013). The present analysis supports the occurrence of a single duplication event in Ranunculales, most probably after the divergence of the Eupteleaceae (**Supplementary Figure 3D**).

Our analyses are consistent with the results obtained with more extensive taxonomic sampling, which suggested that two successive duplications had taken place in the CIN clade after the divergence of Lycophytes and Euphyllophytes (Liu et al., 2019; Lan and Qin, 2020). However, a study with an increased sampling of non-flowering plants has recently questioned the common ancestry of the CIN1 and CIN2 clades (Wang et al., 2022). We found seven CIN1 paralogs in *Amborella*, suggesting several species specific duplications; by contrast, there is only one *Amborella* sequence in each of the two CIN2 clades. A reduced complexity in terms of paralog number was observed in early diverging eudicots (3 sequences in *Aquilegia* and *Nigella*, 5 in *Nelumbo*) compared with the representatives of the core eudicots (6 in *Vitis*, and 8 in *Arabidopsis*) and monocots (8 in *Oryza*). Indeed, no duplications were observed in any of the three CIN sub-clades in Ranunculales (except possible species specific duplications, **Supplementary Figures 3A-C**), while two paralogs were found in the other early diverging eudicot *N. nucifera* in both CIN2 sub-clades. The different number of paralogs among taxa could suggest these are under different functional constraints, but this remains to be explored.

The so-called jaw-TCP genes identified in *Arabidopsis* (AtTCP2/24, AtTCP3/4/10) (Danisman et al., 2013) and other angiosperm species belonged to the CIN1 and CIN2b clades. These genes are specifically targeted by miR319 (Palatnik et al., 2003). No miR targeting site was found in the TCP genes of *Marchantia polymorpha*, *Physcomitrium patens* or *Selaginella mollendorfii*, suggesting that this mode of regulation of TCP genes has evolved after the divergence of Lycophytes (Lan and Qin, 2020), prior to the first duplication of the CIN clade, then lost in the CIN2a clade. In Ranunculales, two alternative variants of the miR targeting site existed, one in CIN1 and the other one in CIN2b, suggesting differential affinity for the miR possibly impacting gene regulation. In addition, the conceptual translation in these regions resulted in different amino acid motifs (QRGPLQSS which is part of ${\text{M}}_{\text{R}}^{\text{II}}9$ in CIN1, and NRGTLQSN in CIN2b), which may impact protein function. The R domain, first described in CYC/TB1 sequences, was present in CIN2b and rarely in CIN2a sequences. Because the R domain is also observed in class II *Physcomitrium* and *Selaginella* sequences (Floyd and Bowman, 2007; Liu et al., 2019), the most parsimonious hypothesis is that this domain is ancestral in class II sequences and was lost in the CIN1 lineage, and independently in many CIN2a sequences.

### TCP gene expression patterns in *N. damascena* are differentiated for RanaCYL and more ubiquitous for RanaCIN and class I genes

The purpose of the analysis was to reveal trends in the expression patterns of TCP genes in aerial organs of *N. damascena*, to guide future trait, organ or tissue specific investigations. We found that all *N. damascena* TCP genes, with the notable exception of the *NdCYL* genes (in the CYC/TB1 clade), were expressed in all organs examined here (**Figure 5**), including at the very early stages of floral development, as revealed in our previous quantitative transcriptomic analysis (Deveaux et al., 2021). Such ubiquitous patterns suggest potential roles in various morphogenetic processes.

CYC/TB1 genes are known to be involved in the control of flower development, plant architecture and shoot branching (Luo et al., 1996; Doebley et al., 1997; Takeda et al., 2003; Kebrom et al., 2006; Kebrom et al., 2012). In *Arabidopsis*, *AtBRANCHED1* (*AtBRC1* a.k.a. *AtTCP18*) and to a lesser extent *AtBRC2* (*AtTCP12*) play a role in shoot branching, in concert with hormonal signals (Aguilar-Martínez et al., 2007; Seale et al., 2017). A similar role has been shown for *AtBRC1* homologs in pea and tomato (Martín-Trillo et al., 2011; Braun et al., 2012). Similarly, in the Papaveraceae species *Eschscholzia californica*, the *CYL1* paralog plays a role in the repression of axillary bud development with a possible redundant contribution of *CYL2*, suggesting that the role of CYC/TB1 genes in branching is conserved among angiosperms (Zhao et al., 2018). After being first characterized in *Antirrhinum majus* for their role in the establishment of zygomorphy (Luo et al., 1996), CYC/TB1 genes have been for the most part studied as candidates for the repeated origin of bilateral symmetry among angiosperms. Asymmetric expression during flower development has been found to be correlated with bilateral symmetry in many diverse angiosperm clades, and independent recruitement for the establishment of floral zygomorphy had been demonstrated in several groups (reviewed in Hileman, 2014). In Lamiales, preliminary evidence suggested that the zygomorphic developmental program depending on CYC has been coopted “en bloc” early during the diversification of the order, from an ancestral role in carpel development (Sengupta and Hileman 2022). In early diverging eudicots and early diverging angiosperms, expression of CYC/TB1 genes is generally found in most floral organs, whether the flower is monosymmetric or polysymmetric (Damerval et al., 2013; Horn et al., 2014; Zhao et al., 2018; Pabón-Mora et al., 2020). Asymmetric floral expression was observed late in development, suggesting that although CYC/TB1 genes could play a role in the zygomorphic phenotype at anthesis, they are almost certainly not the initial triggers (Damerval et al., 2013; Horn et al., 2014; Jabbour et al., 2014; Citerne et al., 2017; Zhao et al., 2018; Pabón-Mora et al., 2020). In the radially symmetric *E. californica*, both *CYL* genes play a role in petal size in addition to having a role in controlling vegetative architecture (Zhao et al., 2018). The *CYL2* gene plays a specific role in controlling stamen number (Zhao et al., 2018). By contrast, in another Papaveraceae species, *Cysticapnos vesicaria*, no vegetative role was found, but *CYL* gene silencing did affect flower development, including sepal and petal identity and floral symmetry (Zhao et al., 2018). In Ranuculaceae, *CYL* paralogs were expressed in floral buds, being either uniformly or not expressed in the perianth organs of actinomorphic species (Jabbour et al., 2014). Our expression data refined these results in *N. damascena*. *NdCYL1* and *NdCYL2* have contrasted expression patterns in the floral organs at the developmental stage studied here, *NdCYL1* being more expressed in gynoecium and *NdCYL2* in petals and stamens, suggesting subfunctionalization (**Figure 5**). Additional duplications in both RanaCYL lineages occurred in the tribe Delphinieae, which contains all the zygomorphic species in the family. Species specific subfunctionalization was also observed among paralogs in Delphinieae species, some of them being asymmetrically expressed in the perianth (Jabbour et al., 2014). In all, available expression and functional data in Ranunculales reveal much species specific subfunctionalization between CYC/TB1 paralogs, advocating for further comparative analyses to better understand the role of these genes in vegetative and floral architecture and how it has evolved during the diversification of the order.

Most functional data on CIN genes have been obtained in the model species *Arabidopsis thaliana* and *Antirrhinum majus*. These genes appear to be at the crossroads of several hormonal biosynthesis and signaling pathways and as such they are involved in many developmental and biological processes (reviewed in Nicolas and Cubas, 2016a; Nicolas and Cubas, 2016b; Sarvepalli and Nath, 2018; Lan and Qin, 2020; Rath et al., 2022). In particular, CIN genes are involved in the control of leaf and petal development and cellular differentiation (Nath et al., 2003; Crawford et al., 2004; Koyama et al., 2011; Das Gupta et al., 2014; Huang and Irish, 2015; Huang and Irish, 2016; Nicolas and Cubas, 2016b; van Es et al., 2018; Chen et al., 2018). Less knowledge has been gained on the function of class I proteins, possibly be due to the difficulty in obtaining altered phenotypes from single gene mutants, suggesting a functional redundancy of these proteins. Additionally, in *Arabidopsis*, class I and CIN genes share several targets and act in the same pathways and processes, either redundantly or antagonistically (Kieffer et al., 2011; Uberti-Manassero et al., 2012; Danisman et al., 2012; Aguilar-Martínez and Sinha, 2013). Expression patterns of CIN and class I genes, and in some cases their functional role in organ development, have been recorded in a few non model species (Ori et al., 2007; Ikeuchi et al., 2013; Lin et al., 2016; Madrigal et al., 2017; Zhao et al., 2020, for review Lan and Qin, 2020). In Ranunculales, the CIN1 clade gene *AqTCP4* has been shown to be required for the proper development of the spurred petals of *Aquilegia coerulea* (Yant et al., 2015). In *Nigella damascena*, the three *NdCIL*s and the nine class I genes were found to be expressed in both the sepals and the elaborate petals as well as in the young floral bud. Both CIN2 paralogs (*NdTCP2* and *NdTCP5*) appeared weakly expressed in stamens compared with other floral organs. The expression pattern of *NdTCP15-2* was different from that of its close paralog *NdTCP15-1*, with a lower expression in the stamens and perianth organs compared with the gynoecium in the former, suggesting sub-functionalization. Overall, we observed quite limited differentiation in the expression patterns of CIN and class I TCP genes in our data. It is worth noting that we did not analyze roots and fruits, which may reveal specific patterns. In the aerial organs at least, functional redundancy could be common among these genes, as has been noticed in *Arabidopsi*s. These seemingly redundant patterns advocate for more detailed analyses examining the fine tuning of TCP gene expression during developmental processes of interest in *Nigella damascena*, such as flower or leaf development, and additional functional studies to determine the role of specific genes. This should be enlightening from an evo-devo point of view, as many similar traits have evolved independently in Ranunculales and core eudicots species.

## Data availability statement

The datasets presented in this study can be found in online repositories. The names of the repository/repositories and accession number(s) can be found below: The transcriptomic data are deposited in the CATdb repository http://tools.ips2.u-psud.fr/CATdb, under the accession ngs2015_16_ranunculaceae and are accessible in the dataverse INRAE at https://doi.org/10.57745/2G1VCP. The sequences and alignments are deposited in the Dryad repository and are accessible at https://doi.org/10.5061/dryad.zcrjdfngw. Nigella TCP sequences are deposited in Genbank with accession numbers OP493852-65.

## Author contributions

CD, YD, NCS, FJ, and SN designed the study, CD, CC, MLG, YD performed the molecular work, LS-T and JC produced the RNA-seq data, VB and ED performed the bioinformatic work, CD and CC performed the phylogenetic analyses, and all authors contributed to writing the manuscript. All authors contributed to the article and approved the submitted version.

## Funding

The project received financial support from the Institut Diversité, Ecologie et Evolution du Vivant (IDEEV AAP2013 and AAP2015).

## Acknowledgments

The GQE-Le Moulon and Institute of Plant Sciences Paris-Saclay benefit from the support of the LabExSaclay Plant Sciences-SPS (ANR-10-LABX-0040-SPS). The authors acknowledge Alioune Badara Ndiaye for help with the molecular work, Adrien Falce for helping with the OrthoFinder method, and Hélène Citerne for constructive comments on the manuscript.

## Conflict of interest

The authors declare that the research was conducted in the absence of any commercial or financial relationships that could be construed as a potential conflict of interest.

## Publisher’s note

All claims expressed in this article are solely those of the authors and do not necessarily represent those of their affiliated organizations, or those of the publisher, the editors and the reviewers. Any product that may be evaluated in this article, or claim that may be made by its manufacturer, is not guaranteed or endorsed by the publisher.

## Supplementary material

Supplementary Table 1 List of primers used.

Supplementary Table 2 TCP gene identifier and abbreviated name for the six angiosperm species included in the phylogenetic reconstruction of the TCP gene family.

Supplementary Table 3
**(A, B)** Class I and class II TCP gene identifier and abbreviated name for all Ranunculales sequences included in this study.

Supplementary Table 4 PCR conditions for the amplification of TCP genes in *N. damascena.*

Supplementary Table 5
**(A, B)** Conserved protein motifs detected in the six angiosperm species plus *N. damascena* (**A**: class I TCP proteins; **B**: class II TCP proteins). The motif order detected on the sequences in the seven species are first indicated. The logos of the motifs are shown in the image, and the correspondence between the motifs detected in the seven species and in the Ranunculales analyses are indicated. Partial overlap of the motif sequences detected in the two analyses is observed in some cases.

Supplementary Figure 1
**(A-F)** Phylogeny of coding nucleotide sequences of class I TCP genes in Ranunculales for each of the six major groups identified in the angiosperm TCP class I phylogeny separately. Phylogenetic trees were rooted with *Nelumbo* (or *Vitis* for RanTCP22) sequences belonging to the corresponding groups in the complete repertoire analysis. Trees were reconstructed using PhyML and branch supports are a-LRT. Regions where primary homology was uncertain were discarded. Reconstructions were based on 909 nucleotide positions out of 1,434 (RanTCP7), 843 out of 1,788 (RanTCP22), 606 out of 876 (RanTCP11), 876 out of 1,401 (RanTCP20), 1,569 out of 1,866 (RanTCP9) and 1,368 out of 1,965 (RanTCP15). Families were color-coded as follows: Eupteleaceae: brown; Lardizabalaceae: khaki green; Berberidaceae: green; Ranunculaceae: pink; Papaveraceae: blue.

Supplementary Figure 2 Sequence logos of the 10 amino acid motifs detected in 145 class I TCP proteins from Ranunculales using MEME.

Supplementary Figure 3
**(A-D)** Phylogeny of coding nucleotide sequences of class II TCP genes in Ranunculales for each subclade. Phylogenetic trees were rooted with the *Nelumbo* sequences used in the complete repertoire analysis. Trees were reconstructed using PhyML and branch supports are a-LRT. Regions where primary homology was uncertain were discarded. Reconstructions were based on 1,716 nucleotide positions out of 3,096 (RanaCIL1), 1,665 out of 1,677 (RanaCIL2a), and 423 out of 2,256 (RanaCYL). Families were color-coded as follows: Eupteleaceae: brown; Circeasteraceae: yellow; Lardizabalaceae: khaki green; Menispermaceae: dark blue; Berberidaceae: green; Ranunculaceae: pink; Papaveraceae: blue.

Supplementary Figure 4 Sequence logos of the 10 amino acid motifs detected in 178 class II TCP proteins from Ranunculales using MEME.

Supplementary Figure 5
**(A)** Picture of a *Nigella damascena* plant, showing the different organs collected for expression analysis. **(B)** Blooming flower with floral organs indicated.

Supplementary Figure 6 Comparison of the expression patterns of TCP gene in *Nigella damascena* in the three biological replicates. **Figure 5** corresponds to R3.
