# Supplementary material for: Evolutionary analyses and expression patterns of TCP genes in Ranunculales
Source: Front Plant Sci. 2022 Dec 1;13:1055196. doi: 10.3389/fpls.2022.1055196 (PMC9752903; doi:10.3389/fpls.2022.1055196)
Supplement: Supplementary Figure 1 — (A-F) Phylogeny of coding nucleotide sequences of class I TCP genes in Ranunculales for each of the six major groups identified in the angiosperm TCP class I phylogeny separately. Phylogenetic trees were rooted with Nelumbo (or Vitis for RanTCP22) sequences belonging to the corresponding groups in the complete repertoire analysis. Trees were reconstructed using PhyML and branch supports are a-LRT. Regions where primary homology was uncertain were discarded. Reconstructions were based on 909 nucleotide positions out of 1,434 (RanTCP7), 843 out of 1,788 (RanTCP22), 606 out of 876 (RanTCP11), 876 out of 1,401 (RanTCP20), 1,569 out of 1,866 (RanTCP9) and 1,368 out of 1,965 (RanTCP15). Families were color-coded as follows: Eupteleaceae: brown; Lardizabalaceae: khaki green; Berberidaceae: green; Ranunculaceae: pink; Papaveraceae: blue. [file DataSheet_1.pdf]

A. RanTCP7

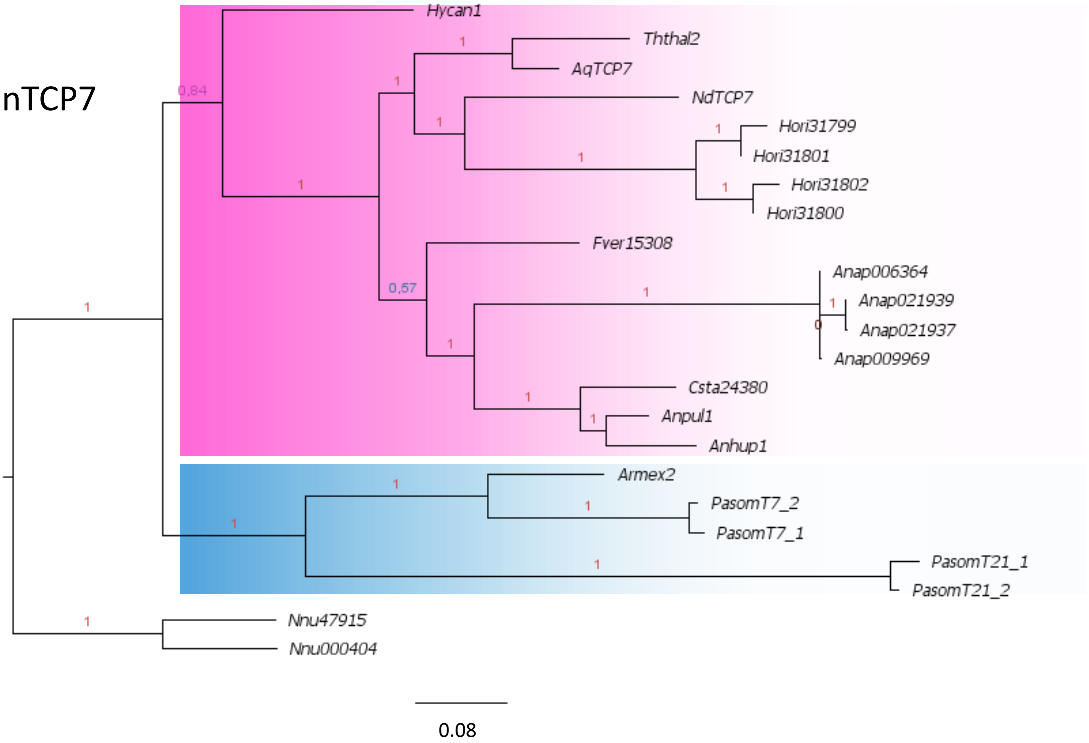

B. RanTCP22

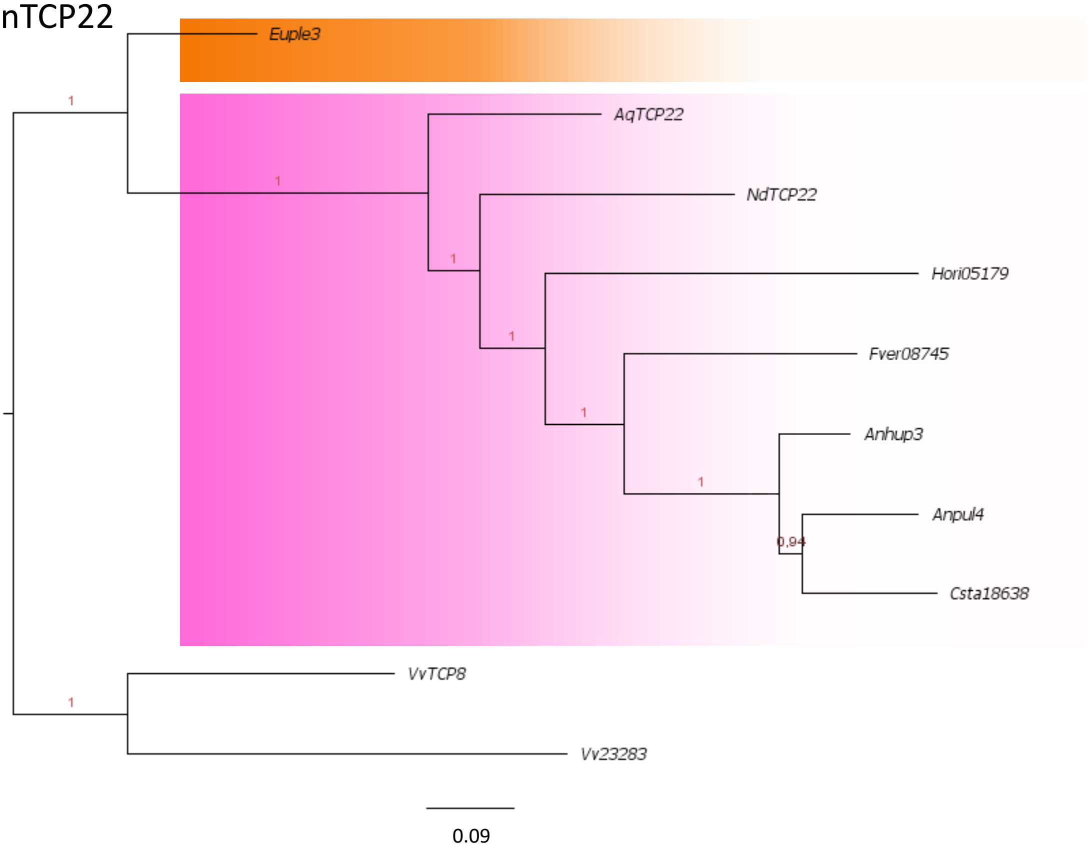

C. RanTCP11

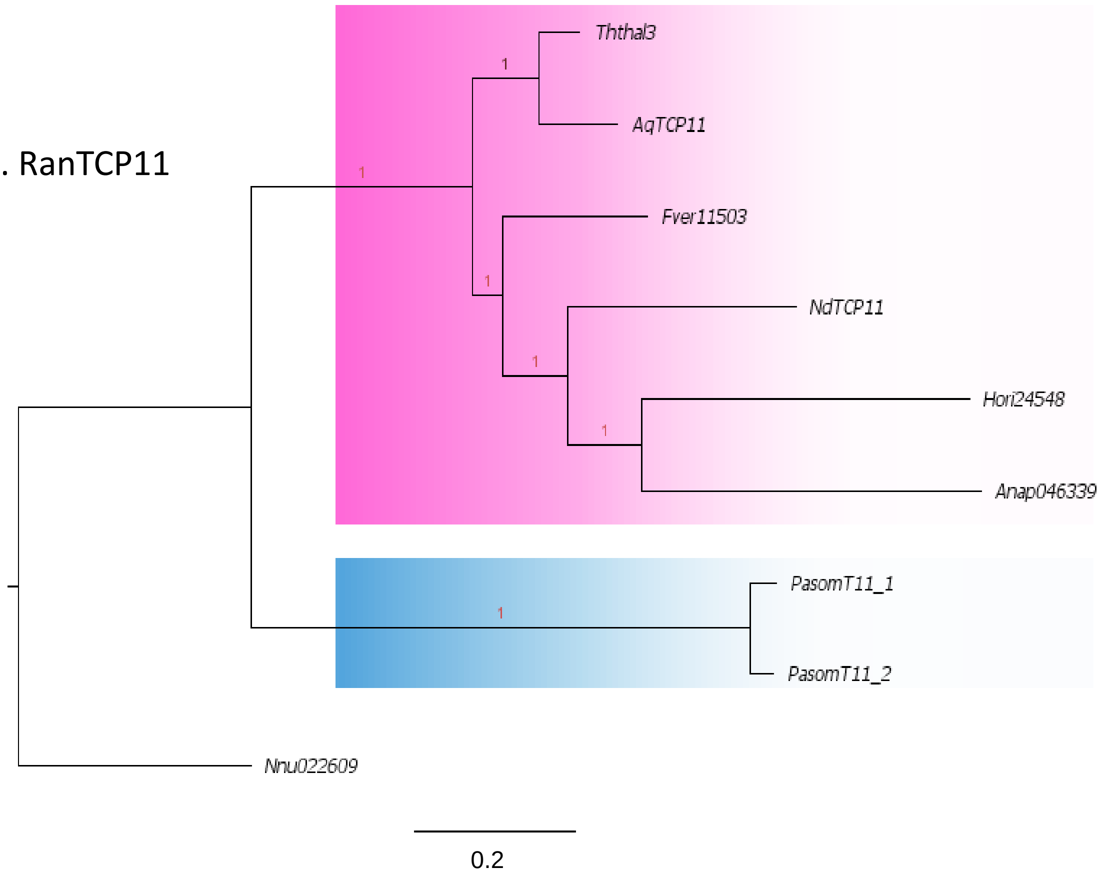

D. RanTCP20

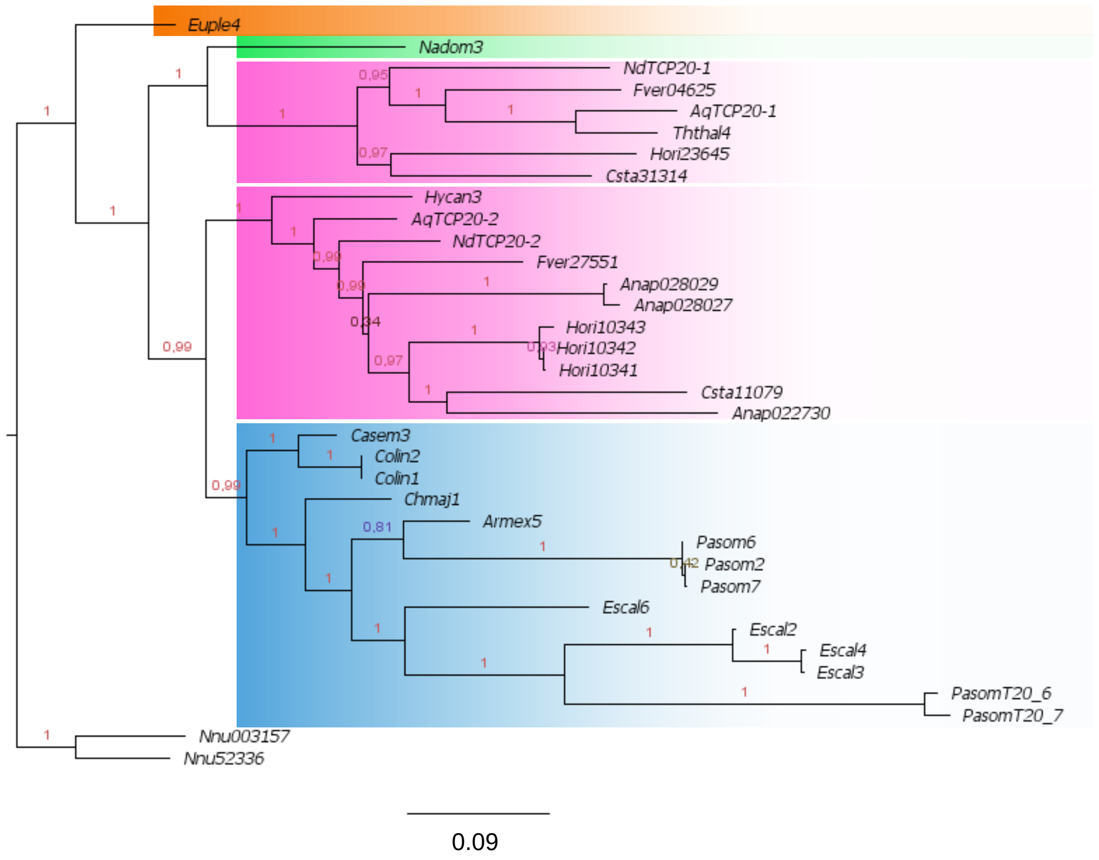

E. RanTCP9

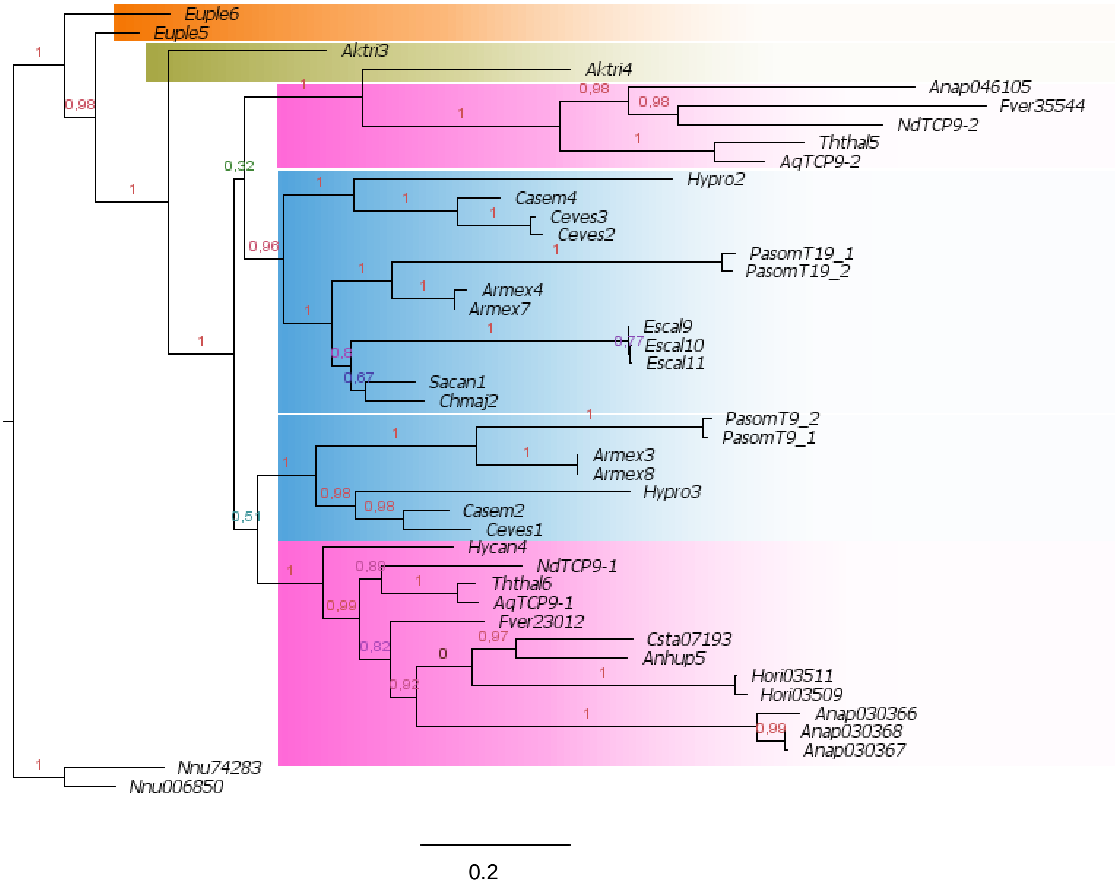

F. RanTCP15

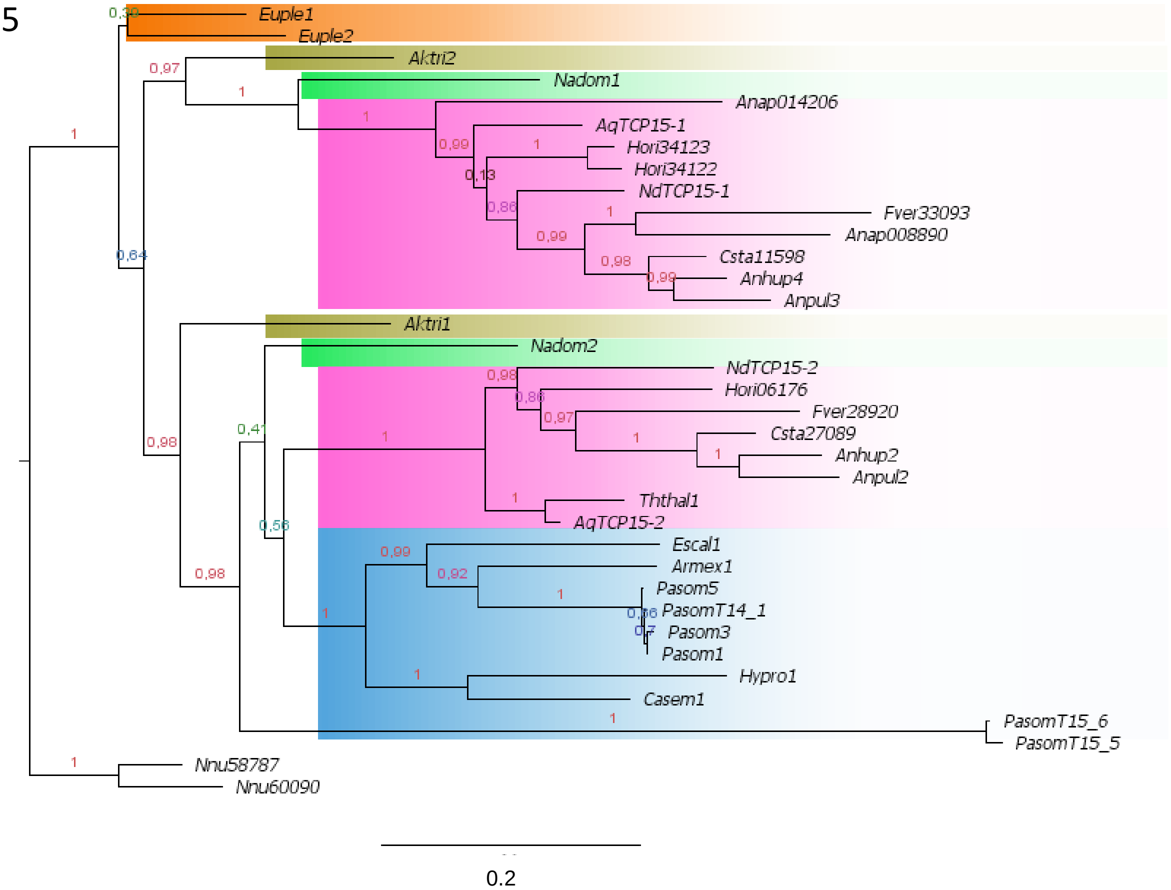

Supplementary Figure 1A-F.

MEME motif in Ranunculales Class I sequences

M_R^I1

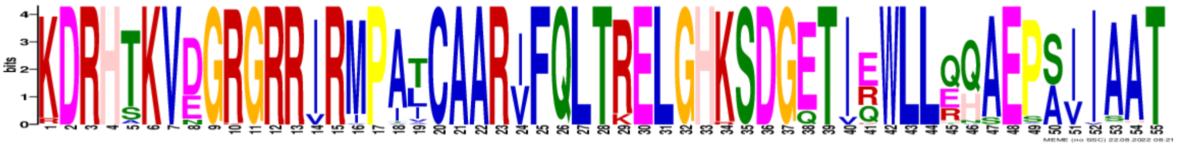

M_R^I2

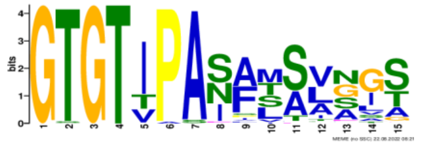

M_R^I3

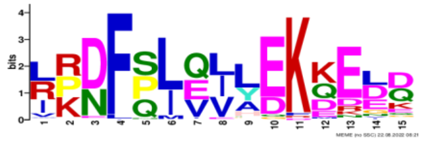

M_R^I4

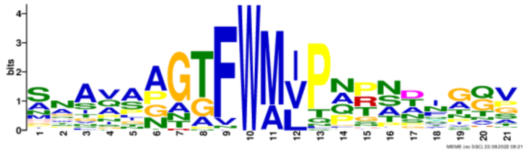

M_R^I5

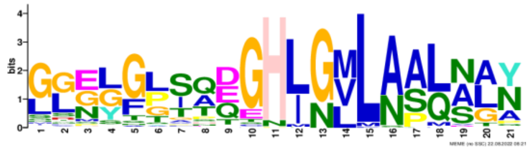

M_R^I6

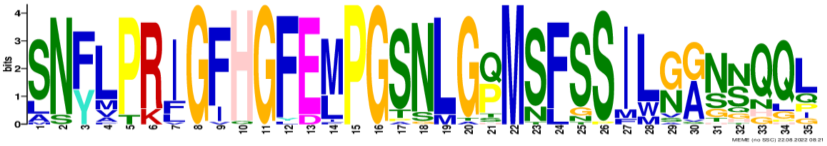

M_R^I7

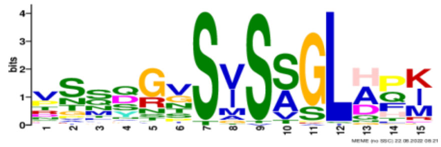

M_R^I8

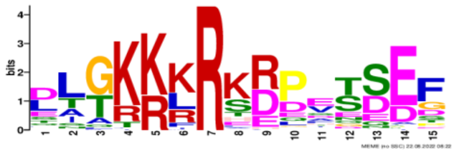

M_R^I9

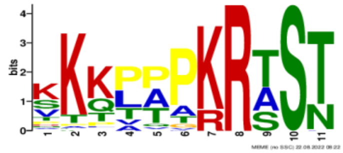

M_R^I10

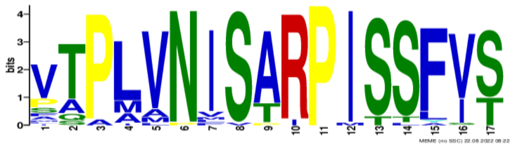

Supplementary Figure 2.

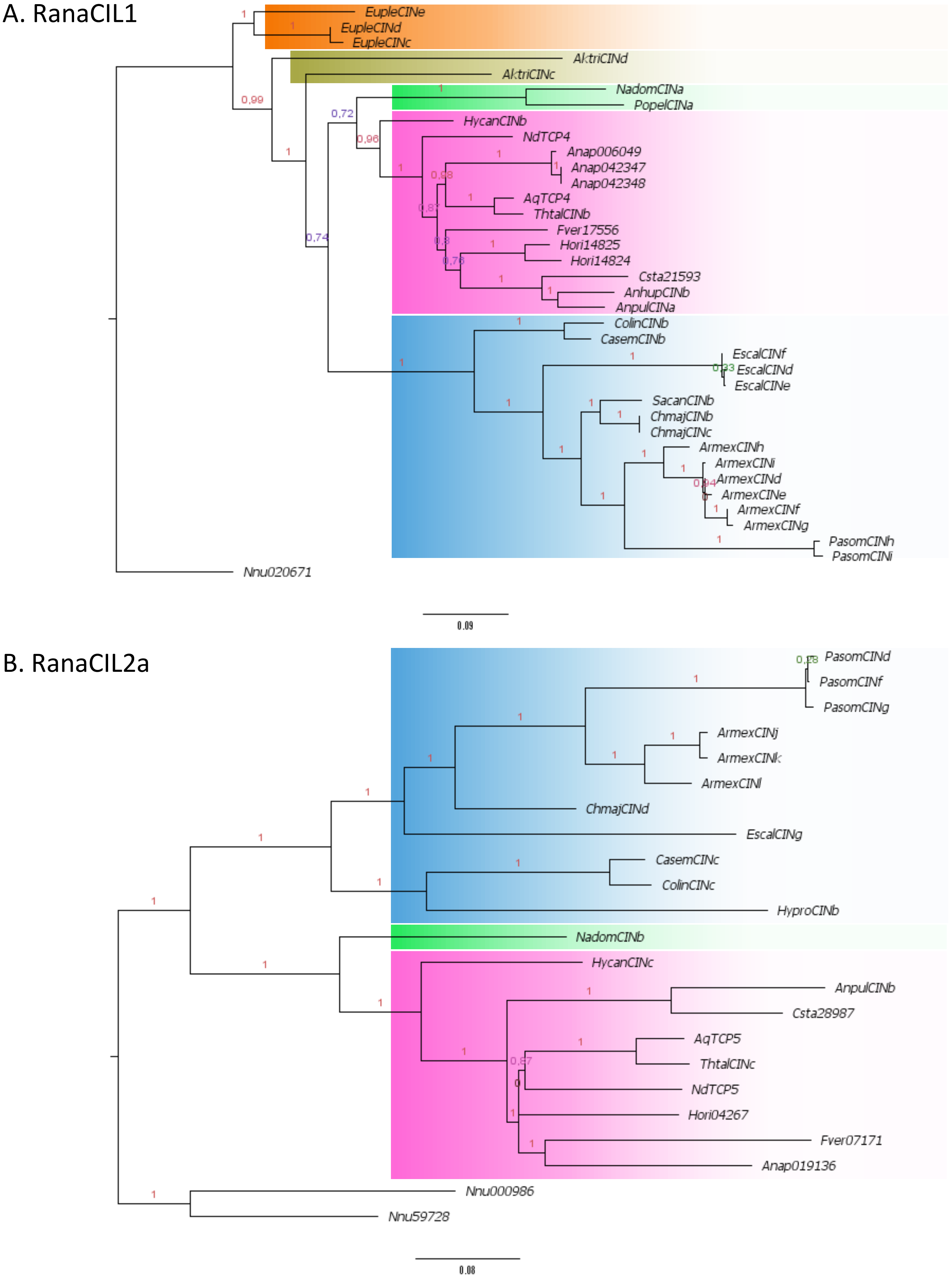
A. RanaCIL1
EupleCINe
EupleCINd
EupleCINc
AktriCINd
AktriCINc
NadomCINa
PopelCINa
HycanCINb
NdTCP4
Anap006049
Anap042347
Anap042348
AqTCP4
ThtalCINb
Fver17556
Hori14825
Hori14824
Csta21593
AnhupCINb
AnpulCINa
ColinCINb
CasemCINb
EscalCINf
EscalCINd
EscalCINe
SacanCINb
ChmajCINb
ChmajCINc
ArmexCINh
ArmexCINi
ArmexCINd
ArmexCINe
ArmexCINf
ArmexCINg
PasomCINh
PasomCINi
Nnu020671
0.09
B. RanaCIL2a
PasomCINd
PasomCINf
PasomCINg
ArmexCINj
ArmexCINk
ArmexCINl
ChmajCINd
EscalCINg
CasemCINc
ColinCINc
HyproCINb
NadomCINb
HycanCINc
AnpulCINb
Csta28987
AqTCP5
ThtalCINc
NdTCP5
Hori04267
Fver07171
Anap019136
Nnu000986
Nnu59728
0.08

C. RanaCIL2b

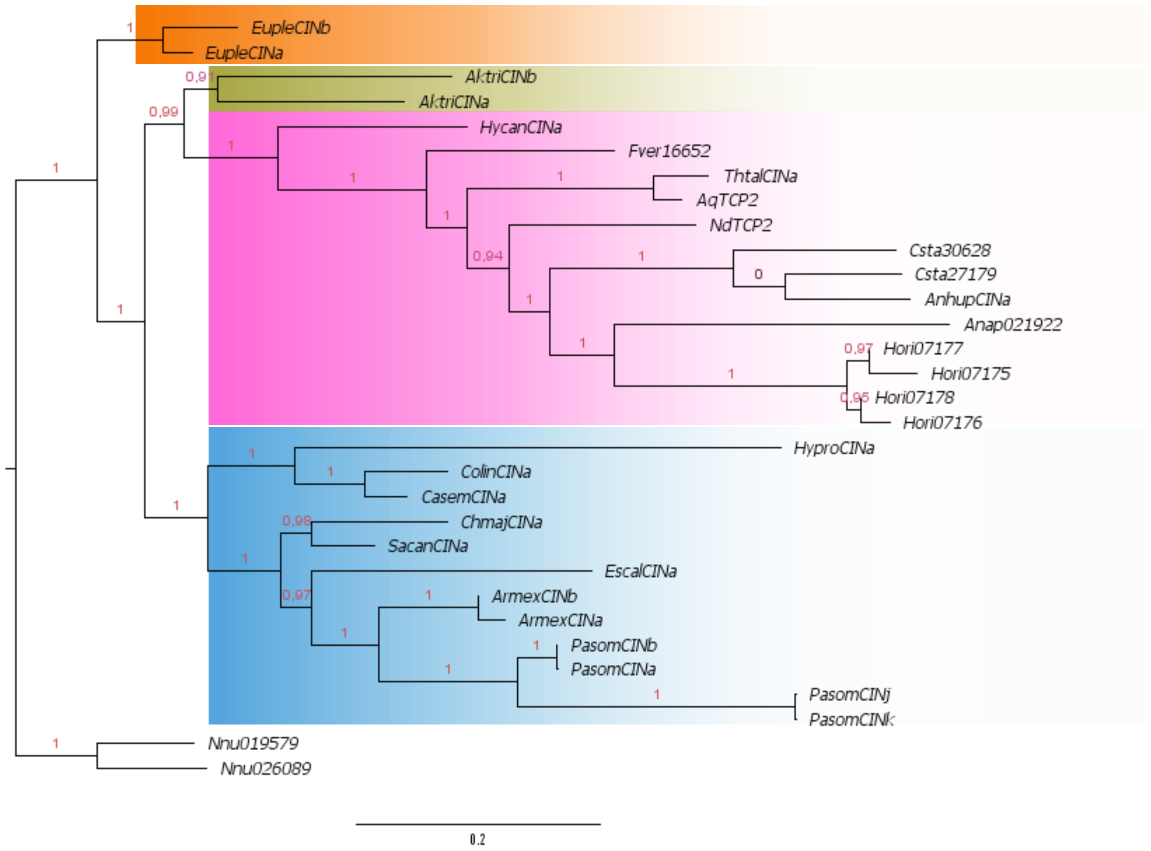
EupleCINb
EupleCINa
AktriCINb
AktriCINa
HycanCINa
Fver16652
ThtalCINa
AqTCP2
NdTCP2
Csta30628
Csta27179
AnhupCINa
Anap021922
Hori07177
Hori07175
Hori07178
Hori07176
HyproCINa
ColinCINa
CasemCINa
ChmajCINa
SacanCINa
EscalCINa
ArmexCINb
ArmexCINa
PasomCINb
PasomCINa
PasomCINj
PasomCINk
Nnu019579
Nnu026089
0.2

D. RanaCYL

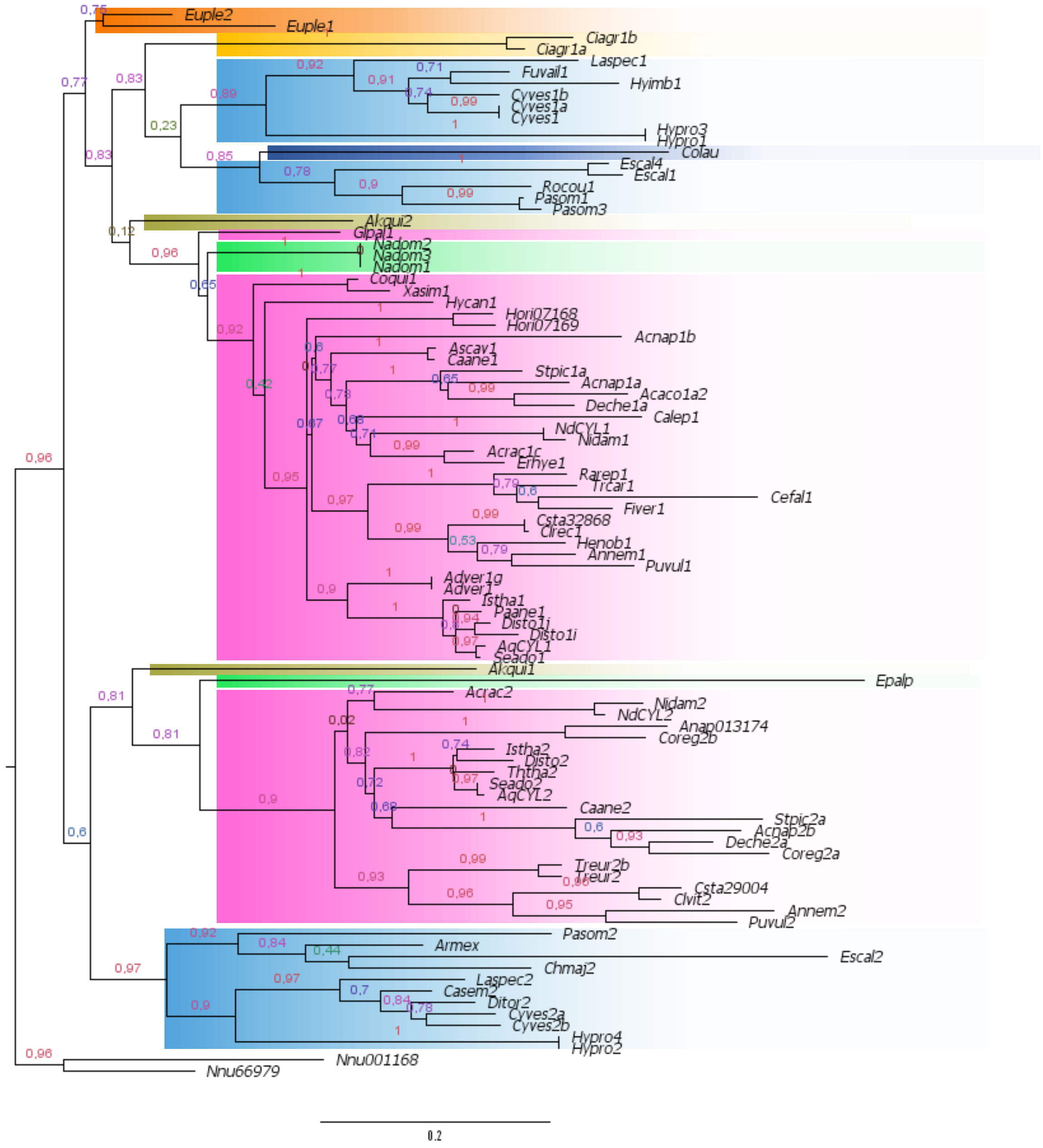

Supplementary Figure 3A-D.

MEME motif in Ranunculales Class II sequences

$M_R^{II}1$ (TCP domain)

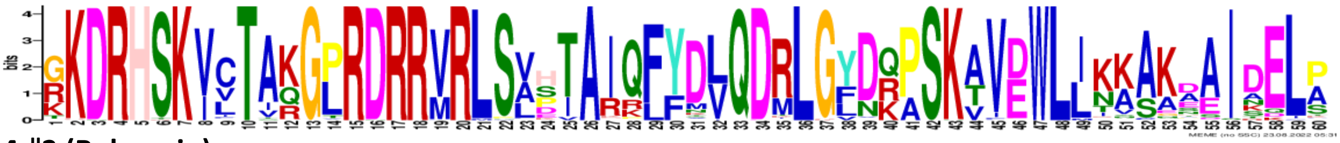

$M_R^{II}2$ (R domain)

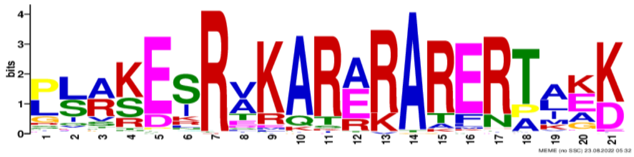

$M_R^{II}3$ (ECE motif)

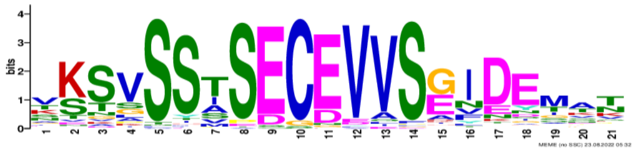

$M_R^{II}4$

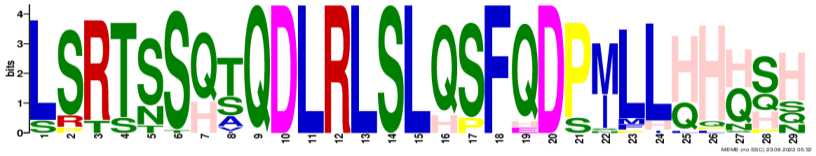

$M_R^{II}5$

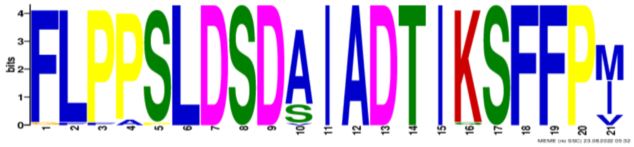

$M_R^{II}6$

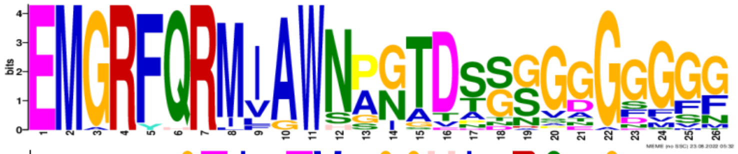

$M_R^{II}7$

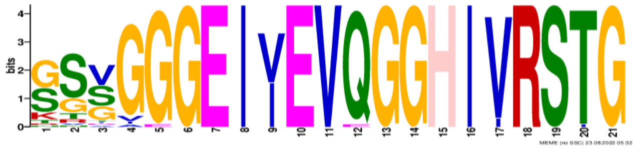

$M_R^{II}8$

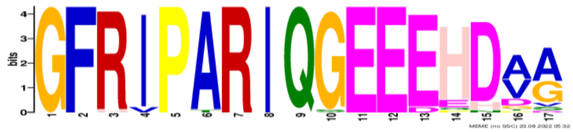

$M_R^{II}9$

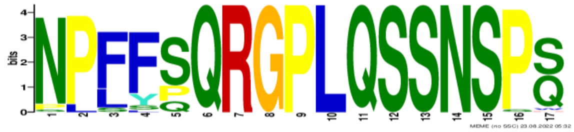

$M_R^{II}10$

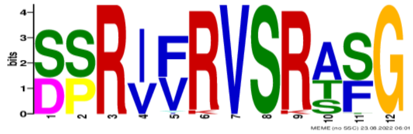

Supplementary Figure 4.

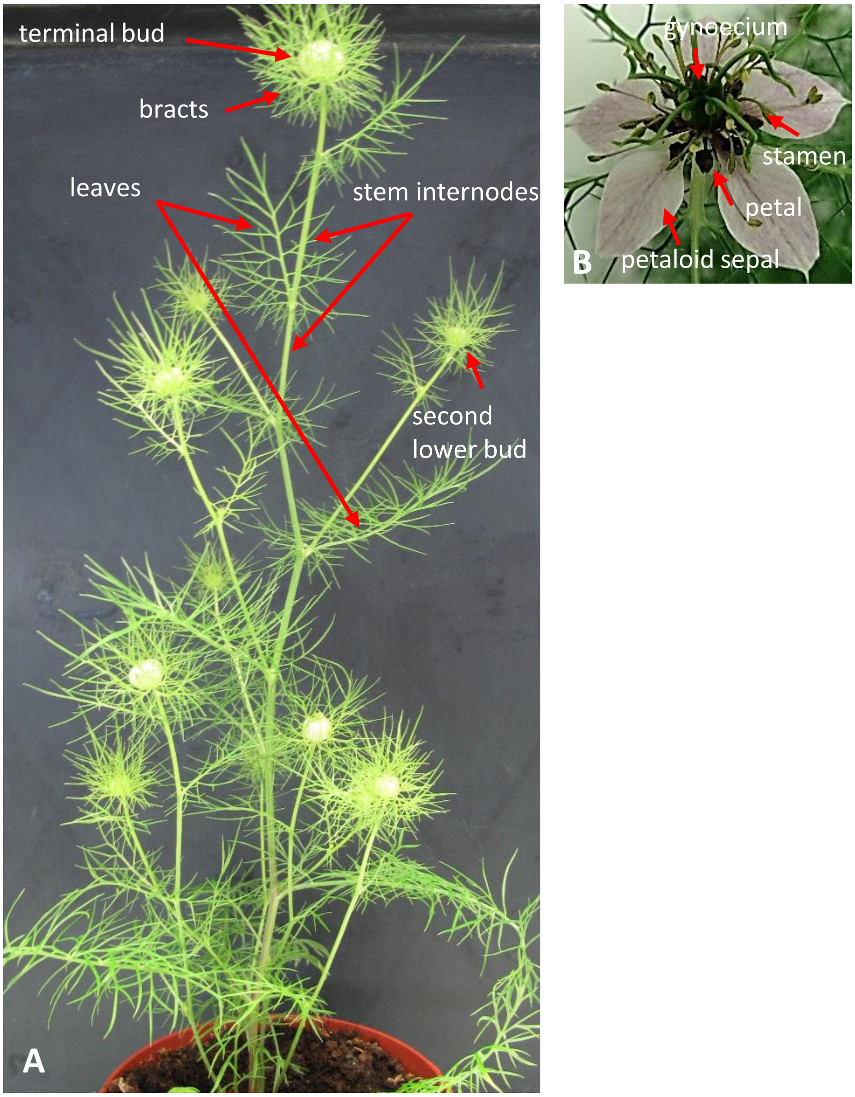

Supplementary Figure 5.

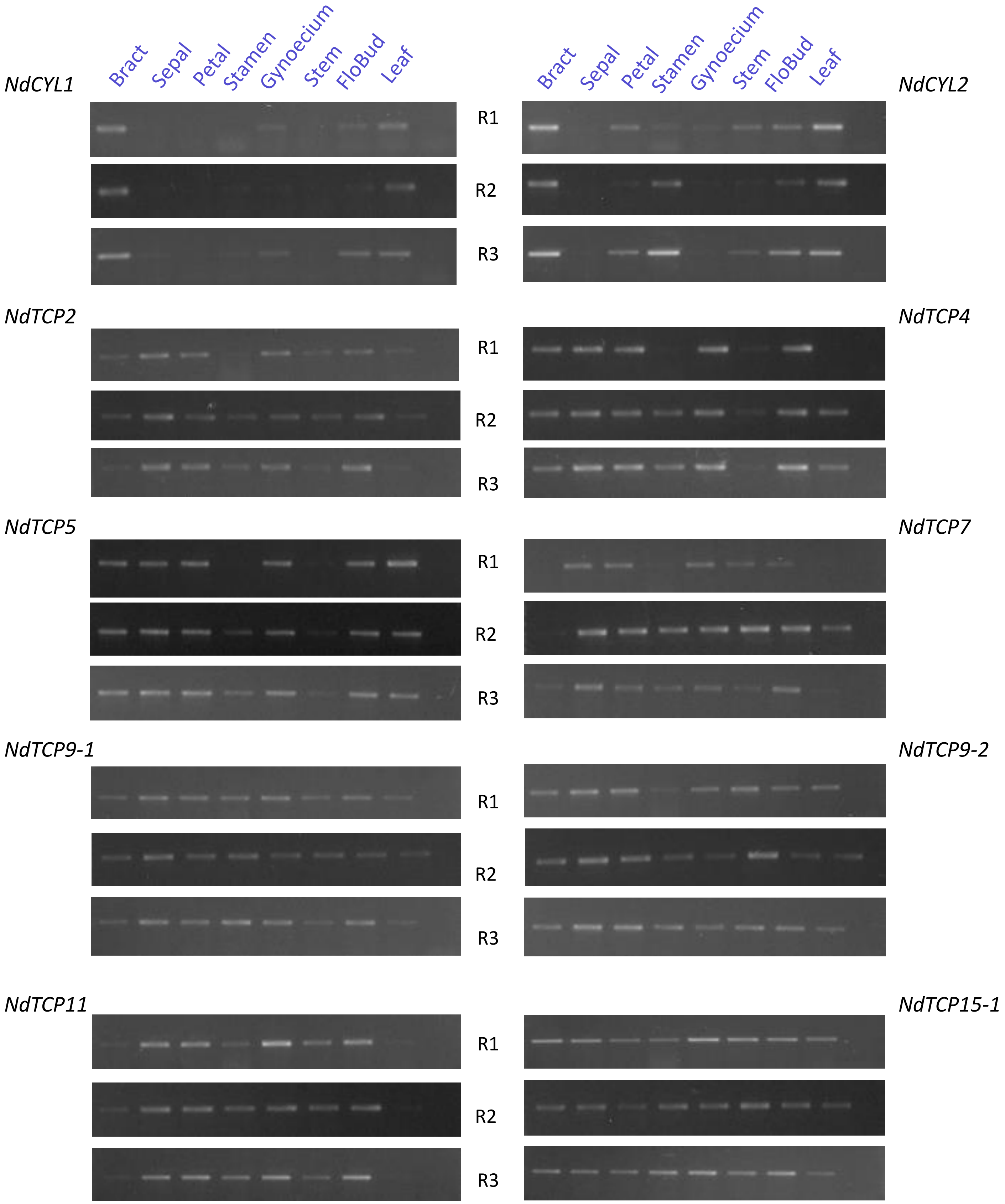

NdCYL1
NdCYL2
Bract
Sepal
Petal
Stamen
Gynoecium
Stem
FloBud
Leaf
R1
R2
R3
NdTCP2
NdTCP4
NdTCP5
NdTCP7
NdTCP9-1
NdTCP9-2
NdTCP11
NdTCP15-1

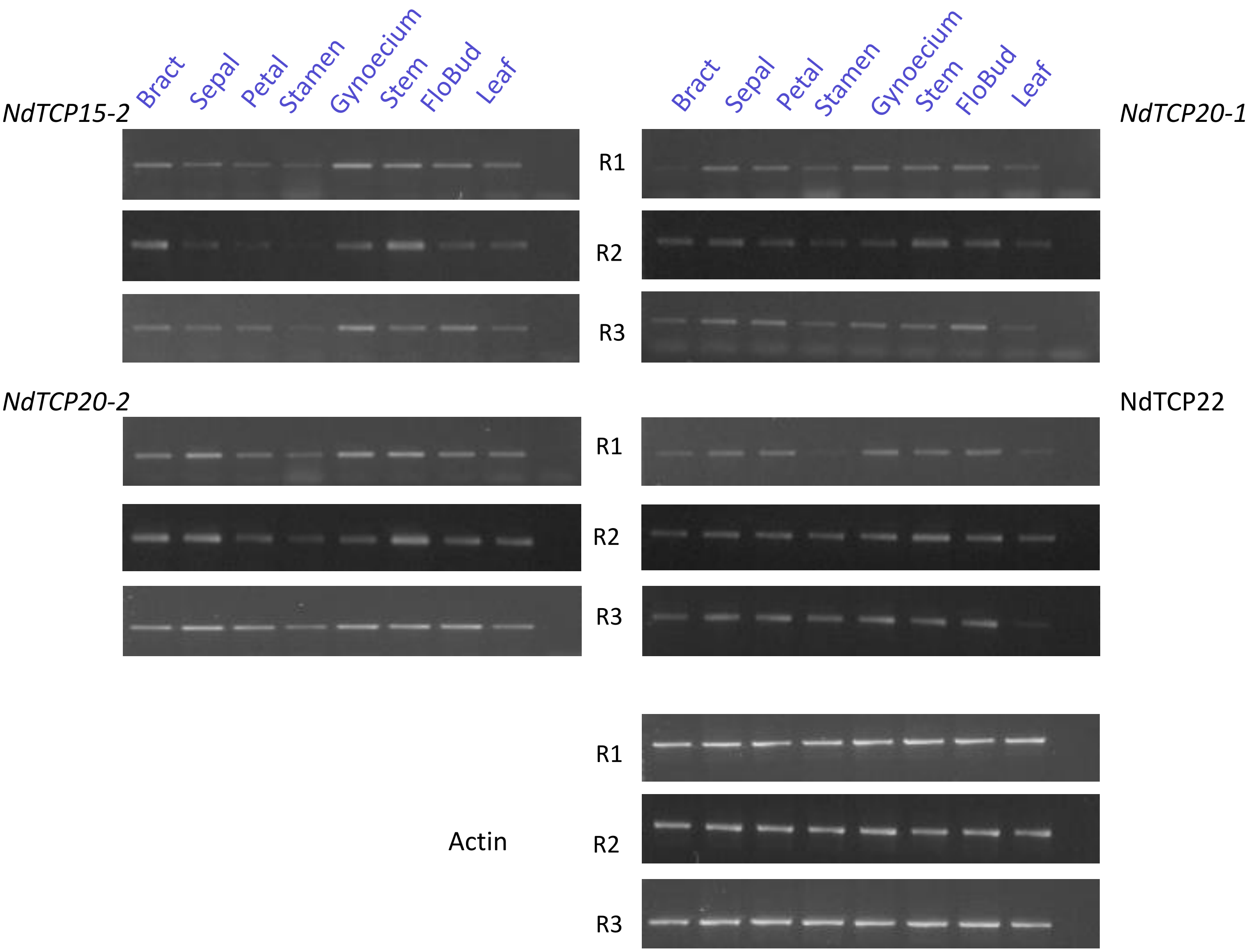

Supplementary Figure 6.
